# Development and Characterisation of a New Patient-Derived Xenograft Model of AR-Negative Metastatic Castration-Resistant Prostate Cancer

**DOI:** 10.3390/cells13080673

**Published:** 2024-04-12

**Authors:** Daniel J. Turnham, Manisha S. Mullen, Nicholas P. Bullock, Kathryn L. Gilroy, Anna E. Richards, Radhika Patel, Marcos Quintela, Valerie S. Meniel, Gillian Seaton, Howard Kynaston, Richard W. E. Clarkson, Toby J. Phesse, Peter S. Nelson, Michael C. Haffner, John N. Staffurth, Helen B. Pearson

**Affiliations:** 1 The European Cancer Stem Cell Research Institute, School of Biosciences, Cardiff University, Hadyn Ellis Building, Cardiff CF24 4HQ, UK; 2 Cancer Research UK Scotland Institute, Glasgow G61 1BD, UK; 3 Division of Human Biology and Clinical Research, Fred Hutchinson Cancer Center, Seattle, WA 98109, USA; 4 Division of Cancer and Genetics, School of Medicine, Cardiff University, Cardiff CF14 4XN, UK; 5 Department of Urology, Cardiff and Vale University Health Board, University Hospital of Wales, Cardiff CF14 4XW, UK; 6 The Peter Doherty Institute for Infection and Immunity, The University of Melbourne, Melbourne, VIC 3000, Australia; 7 Department of Laboratory Medicine and Pathology, University of Washington, Seattle, WA 98195, USA; 8 Department of Urology, University of Washington, Seattle, WA 98195, USA; 9 Department of Medicine, University of Washington, Seattle, WA 98195, USA

**Keywords:** patient-derived xenograft (PDX), double-negative prostate cancer (DNPC), castration-resistant prostate cancer (CRPC), neuroendocrine (NE), androgen receptor (AR)

## Abstract

As the treatment landscape for prostate cancer gradually evolves, the frequency of treatment-induced neuroendocrine prostate cancer (NEPC) and double-negative prostate cancer (DNPC) that is deficient for androgen receptor (AR) and neuroendocrine (NE) markers has increased. These prostate cancer subtypes are typically refractory to AR-directed therapies and exhibit poor clinical outcomes. Only a small range of NEPC/DNPC models exist, limiting our molecular understanding of this disease and hindering our ability to perform preclinical trials exploring novel therapies to treat NEPC/DNPC that are urgently needed in the clinic. Here, we report the development of the CU-PC01 PDX model that represents AR-negative mCRPC with PTEN/RB/PSMA loss and *CTNN1B*/*TP53/BRCA2* genetic variants. The CU-PC01 model lacks classic NE markers, with only focal and/or weak expression of chromogranin A, INSM1 and CD56. Collectively, these findings are most consistent with a DNPC phenotype. Ex vivo and in vivo preclinical studies revealed that CU-PC01 PDX tumours are resistant to mCRPC standard-of-care treatments enzalutamide and docetaxel, mirroring the donor patient’s treatment response. Furthermore, short-term CU-PC01 tumour explant cultures indicate this model is initially sensitive to PARP inhibition with olaparib. Thus, the CU-PC01 PDX model provides a valuable opportunity to study AR-negative mCRPC biology and to discover new treatment avenues for this hard-to-treat disease.

## 1. Introduction

Prostate cancer is the second most diagnosed cancer in men, accounting for 14.1% of new cases globally and >375,000 deaths annually [1]. The major cause of death is the development of metastatic castrate-resistant prostate cancer (mCRPC) following progression on androgen/androgen receptor (AR)-targeted therapy (ARTT) and the limited efficacy of chemotherapy [2]. NEPC and DNPC are aggressive hard-to-treat subtypes of prostate cancer that are estimated to emerge in 15–25% of patients treated with ARTT [3,4,5]. Although rare cases of de novo NEPC have been reported, treatment-induced NEPC and DNPC are far more common [6]. Treatment-induced NEPC is often identified through the emergence of a small cell prostate carcinoma (SCPC) with either a pure or mixed histopathology as tumours transition from prostate adenocarcinoma towards SCPC [6]. Common hallmarks of NEPC include AR loss, low serum levels of prostate-specific antigen (PSA), and the increased expression of NE markers such as chromogranin A (CHGA), synaptophysin (SYP), insulinoma-associated 1 (INSM1), and the neural cell adhesion molecule CD56 [3,6,7]. Significantly, the absence of AR in NEPC tumours renders ARTT ineffective, and current treatment options for patients with NEPC are limited to platinum-based chemotherapy with poor clinical outcome [8]. DNPC is currently classified as AR negative prostate cancer without overt NE differentiation markers [5]. Similar to NEPC, DNPC has been shown to have a poor overall survival rate [4]. Clinical trials exploring prostate specific membrane antigen (PSMA) and delta-like protein 3 (DLL3) targeted therapies (e.g., Lu^177^-PSMA and Lu^177^-DLL3 based therapies) may prove to be beneficial in PSMA-positive or DLL3-positive NEPC/DNPC, respectively, however most DNPC and NEPC tumours are negative for PSMA and only a subset of NEPC tumours express DLL3 [4,9,10,11,12]. These features highlight the need to increase our molecular understanding of NEPC/DNPC biology to facilitate the identification of new therapeutic strategies to treat this lethal disease [12].

The clinical demand for new effective treatment strategies for NEPC/DNPC is likely to increase over time owing to the widespread use of ARTT, such as enzalutamide and abiraterone acetate, which are associated with their increased occurrence [4,13]. The application of appropriate preclinical models that are both reproducible and representative of the disease observed in the clinic is critical for the discovery of new therapeutic avenues for NEPC/DNPC. Patient-derived xenografts (PDXs) represent the closest pre-clinical models preceding clinical evaluation, however historically these have been particularly hard to establish for prostate cancer reflecting low take rates and an inability to be serially transplanted [14]. In recent years, a growing number of prostate PDX panels that include several models with mixed prostate adenocarcinoma/NEPC and NEPC histopathologies have been generated [15,16,17]. However, DNPC PDX models are rare [18].

Here we describe the CU-PC01 PDX, a new preclinical AR-negative mCRPC model that can be serially transplanted and cryopreserved. Whole exome sequencing revealed the CU-PC01 model harbours a *TP53* loss of function mutation, β-catenin (*CTNNB1*) activating mutation, and a previously unreported *BRCA2* missense mutation, in concordance with the donor patient mCRPC biopsy. CU-PC01 tumours display DNPC-like clinicopathological features, including the absence of AR, PSA and PSMA, loss of PTEN and RB, and a lack of NE markers, including SYP, neuronal differentiation 1 (NEUROD1), POU class 2 homeobox 3 (POU2F3) and achaete-scute homolog 1 (ASCL1). Only weak, focal positivity for NE markers INSM1 and CD56 was detected, plus rare CHGA-positive cells. We show that the CU-PC01 PDX model is insensitive to both enzalutamide and docetaxel, thus providing a readily available tool for the research community to boost our understanding of this hard-to-treat disease, and to facilitate the development of novel therapeutic strategies for AR-negative mCRPC that benefit patients.

## 2. Materials and Methods

### 2.1. Patient Sample Collection and Processing

In collaboration with the Wales Cancer Bank (WCB), Velindre Hospital, and the University Hospital of Wales (UHW), a metastatic lymph node image-guided needle biopsy was collected (2 × 20 mm) from a mCRPC patient experiencing increased pelvic pain within 3 months of commencing enzalutamide treatment and a recent fall in PSA levels (2.8 ng/mL to 1.0 ng/mL). The biopsy specimen was immediately placed in transport media (previously described in [19], supplemented with 5 μM ROCK inhibitor Y-27637, HelloBio #HB2297) and stored on ice for subsequent processing. A small proportion of the biopsy specimen was snap-frozen for subsequent molecular characterisation or fixed in 4% neutral buffered formalin on ice for 16 h before being paraffin embedded for H&E analysis and immunohistochemistry (IHC) staining. The remainder of the biopsy specimen was transferred to dissection media (previously described in [19], supplemented with 5 μM ROCK inhibitor Y-27637, HelloBio #HB2297), and divided into 2–3 mm^3^ fragments for PDX model propagation within 45 min of receiving the biopsy. The WCB is an ethically approved research tissue bank (Wales Research Ethics Committee reference 21/WA/0234) [20]. Molecular characterisation and PDX model generation was approved through the WCB ethics committee (application 17-014, Wales Research Ethics Committee reference 16/WA/0256) and in accordance with informed written patient consent. Tissue blocks were reviewed by certified pathologists at WCB/UHW and the project adhered to the Human Tissue Authority codes of practice and standards.

### 2.2. CU-PC01 PDX Model Generation and Cryopreservation

Tumour fragments (2–3 mm^3^) were bilaterally implanted subcutaneously into the left and right flanks of male, non-obese diabetic severe combined immunodeficiency interleukin 2-receptor gamma chain knockout (NSG) mice (Charles River, Kent, UK), aged 6–8 weeks old. UV-sterilised testosterone implants were prepared and simultaneously implanted subcutaneously, as previously described [19]. CU-PC01 PDX tumours were serially transplanted when the tumour burden approached ethical size limits. Passages 7 and 8 were transplanted into 8 week-old Athymic Nude male mice (Charles River, Kent, UK). The testosterone pellet was not implanted at passage 8. Up to six 2–3 mm^3^ tumour fragments were cryopreserved in FCS-R cryopreservation media (previously described in the literature [21]) using a CoolCell freezing container (Corning, #432003) at a rate of 1 °C/min at −80 °C for 24 h, and transferred to an LN2 tank for long-term storage. Cryopreserved samples were thawed and transferred to the cryorecovery media (90% *v*/*v* RPMI 1640 (Thermofisher, #21875-059), 10% *v*/*v* heat-inactivated FBS (Sigma #F9665), 25 mM HEPES (Thermofisher #15630106), 10 nM testosterone (Sigma #T1500), and 5 μM ROCK inhibitor Y-27637 (HelloBio, #HB2297) prior to engraftment into 6–8 week-old NSG male mice supplemented with testosterone. All mouse husbandry and experiments were carried out in accordance with UK Home Office regulations and the Animals (Scientific Procedures) Act 1986, and approved by the Cardiff University Animal Welfare Ethical Review Body (AWERB).

### 2.3. DNA Extraction and Whole Exome Sequencing

Snap-frozen tumour specimens were homogenised using the FastPrep24-5G homogeniser (MP Biomedicals, #116005500). DNA was isolated using the Qiagen DNeasy Blood and Tissue Kit following the manufacturer’s instructions (Qiagen, #69504). DNA quality was checked by agarose gel electrophoresis and quantified using the Qubit^®^ DNA Assay Kit in Qubit^®^ 2.0 Fluorometer (Life Technologies, South San Francisco, CA, USA) by Novogene (Cambridge, UK). WES and library construction was performed by Novogene using the Agilent SureSelect Human All Exon V6 Kit (Agilent Technologies, Santa Clara, CA, USA) following the manufacturer’s recommendations and x-index codes were added to the attribute sequences for each sample. In brief, fragmentation was carried out by a hydrodynamic shearing system (Covaris, MA, USA) to generate 180–280 bp fragments. Remaining overhangs were converted into blunt ends via exonuclease/polymerase activities and enzymes were removed. After adenylation of 3′ ends of DNA fragments, adapter oligonucleotides were ligated. DNA fragments with ligated adapter molecules on both ends were selectively enriched in a PCR reaction. Captured libraries were enriched in a PCR reaction to add index tags to prepare for hybridisation. Products were purified using the AMPure XP system (Beckman Coulter, Beverly, CA, USA) and quantified using the Agilent high sensitivity DNA assay on the Agilent Bioanalyzer 2100 system. After cluster generation, the DNA libraries were sequenced using the Illumina NovaSeq 6000 platform (Illumina, San Diego, CA, USA), yielding 150 bp paired-end reads. WES data was quality control checked to remove sequencing artifacts (adapter contamination, low-quality nucleotides and unrecognisable nucleotides) by Novogene (sequencing error rate <0.03%, phred-scaled quality score greater than 20 ≥96.5%, GC% = 47.96–50.55%). Sequencing alignment and variant calling was performed by Novogene; the Burrows–Wheeler Aligner (BWA) v0.7.17 was utilized to map the paired-end clean reads to the human reference genome (hg38). SAMtools v1.8 was used to sort the BAM files [22], Picard v2.18.9 was employed to mark duplicate reads, the ANNOVAR tool was used to annotate variants [23], and GATK was used to detect and filter single nucleotide variants and insertions/deletions (SNPs/INDELs) [24]. Allelic frequency scores were determined as heterozygous (AF = 0.5) or homozygous (AF = 1.0) by Novogene. SIFT scores were calculated using dbNSFP version 3.3a, where D (Deleterious, score <= 0.05); T (Tolerated, score > 0.05) [25]. Subsequent analysis was performed using base R functions and the software packages ggalluvial and ComplexHeatmap. 

### 2.4. Gene Signature Enrichment

Gene signature enrichment was performed (observed vs. expected) using the consensus gene list (present in all samples) in R. A null distribution (expected) was calculated by simulating the overlap between the consensus gene set and a random set of genes of the same size as the gene signature 1,000,000 times. An Obs/Exp value was calculated as a ratio, *p* values were calculated by observing how many times the mean null value was greater than the observed overlap (formula: mean(null >= length(ov1)), where ov1 = observed overlap, null = a numeric vector of 1,000,000 random overlaps). To assess genetic alterations in pathways/cellular processes commonly deregulated in prostate cancer, several gene lists were employed to assess androgen receptor (AR) signalling (*n* = 22) [26,27,28], epigenetic regulators (*n* = 12) [29], cell cycle regulators (*n* = 5) [29], the DNA damage repair pathway (*n* = 11) [29], mitogen-activated protein kinase (MAPK) signalling (*n* = 167, Harmonizome GO:0000165) [30], phosphatidyl inositol-3 kinase (PI3K) signalling (*n* = 68) [31], Nuclear factor-kappa B (NF-kB) signalling (*n* = 48) [30,32,33], Wnt signalling (*n* = 70) [34], and NEPC associated genes (*n* = 31) [35,36] (detailed in Supplementary Table S1).

### 2.5. Epstein–Barr Virus (EBV) Detection

Snap-frozen PDX tumour fragments were homogenised using the FastPrep24-5G homogeniser (MP Biomedicals, #116005500) in lysing matrix D tubes and DNA was extracted using the Qiagen AllPrep DNA/RNA/Protein Mini Kit following the manufacturer’s instructions (Qiagen, #80004). A common region of EBV nuclear antigen 1 and 2 (*EBNA*) was detected using the previously published primers *EBNA-2F* 5′-TGGAAACCCGTCACTCTC-3′ and *EBNA-2I* 5′-TAATGGCATAGGTGGAATG-3′ (PCR product = 801 bp) [37]. DNA was amplified using GoTaq G2 Flexi DNA polymerase (Promega, #M7801) according to the manufacturer’s instructions. Cycling conditions: 94 °C for 2 min, 35 cycles of 94 °C for 1 min, 58 °C for 90 s, 72 °C for 4 min, followed by 72 °C for 10 min. Genomic DNA from Raji cells, a human B lymphoblastoid cell line, served as an EBV positive control.

### 2.6. Immunohistochemistry

IHC staining was carried out as described previously [38,39,40] on formalin-fixed, paraffin-embedded (FFPE) 4 µm thick tissue sections. The primary antibodies included: Abcam antibodies ASCL1 1:100 (#ab211327), CK5 1:500 (#ab52635), CK8 1:500 (#ab53280), NEUROD1 1:25 (#ab205300), and SYP 1:500 (#ab32127); Cell Signalling Technology antibodies CD56 1:100 (#99746), Cleaved caspase-3 (CC-3) 1:300 (#9664), HOXB13 1:50 (#90944), PSA 1:50 (#2475), PTEN 1:300 (#9559), phosphorylated eukaryotic translation initiation factor 4E binding protein 1 (p-4EBP1) 1:500 (#2855) and phosphorylated ribosomal protein S6 (p-RPS6) 1:400 (#4858); Sigma antibodies AR 1:250 (#06-680) and human mitochondria (Hu. Mito) 1:200 (#MAB1273); Santa Cruz antibodies INSM1 1:50 (#sc271408) and POU2F3 1:100 (#sc293402); BD Biosciences antibodies; proliferating cell nuclear antigen (PCNA) 1:400 (#610665) and Retinoblastoma protein (RB) 1:100 (#554136); Invitrogen antibody CHGA 1:400 (#PA5-32349); Agilent antibody PSMA 1:20 (#M3620); and Neomarker antibody PTEN 1:200 (#10P03). Prior to primary antibody incubation, antigen retrieval was performed using a microwave pressure cooker (Nordic Ware, Minneapolis, MN, USA #62104) with either citrate buffer pH 6.0 (Generon, Slough, UK, #CBB999: SYP, AR, or Vector Labs #H-3300-250: CD56, ASCL1, PSA, HOXB13), EDTA pH 8.0 (PCNA, CC3), Dako high pH solution (Agilent technologies, Manchester, UK, #S236784-2: CHGA, PTEN, p-4EBP1, p-RPS6, Hu. Mito, CK5, CK8, RB), or Dako target retrieval buffer (Agilent technologies, Manchester, UK, #S169984-2: INSM1, NEUROD1, POU2F3, PSMA). Anti-rabbit (Dako, CA, USA, #E0432) or anti-mouse (Dako, #E0433) biotin-conjugated secondary antibodies were followed by the VectaStain ABC-HRP detection kit (#PK-4000), liquid DAB+ visualisation (Agilent technologies, #K346811-2) and a haematoxylin counterstain (Atom Scientific, Manchester, UK, #RRSP62-D), according to the manufacturer’s instructions. Exceptions included CD56, ASCL1 and NEUROD1 that were detected using the PowerVision anti-rabbit kit (Leica Microsystems, Milton Keynes, UK, #PV6119), POU2F3, PSMA and INSM1 that were detected using PowerVision anti-mouse kit (Leica Microsystems, Milton Keynes, UK, #PV6114) and PSA and HOXB13 that were detected using the UltraVision Quanto detection system (Fisher Scientific, Loughborough, UK, #TL-060-QHD). POU2F3 and PSMA staining also included an additional Biotin-Tyramide SuperBoost Streptavidin kit step (Fisher Scientific #B40931) following the PowerVision secondary antibody incubation.

### 2.7. Protein Isolation and Western Blotting

Protein was isolated from snap-frozen CU-PC01 tumours using RIPA buffer (Universal Biologicals Ltd., Cambridge, UK #39244.01) supplemented with a protease/phosphatase inhibitor cocktail (Cell Signalling Technology, Leiden, Netherlands, #5872S) and homogenised with 1.4 mm ceramic beads (VWR, Leicestershire, UK, #432-0372P) in a FastPrep24-5G homogeniser (MP Biomedicals, CA, USA, #116005500). Protein concentrations were determined using a Bradford assay (Bio-Rad, Watford, UK, #5000205), before equal amounts of protein were separated using 10% Mini-PROTEAN TGX gels (Bio-Rad, #4561034) and transferred onto mini PVDF membranes (Bio-Rad, #1704156) using the trans-blot turbo transfer system (Bio-Rad, #1704150). Membranes were blocked in 5% BSA (Sigma), incubated with the primary antibody overnight at 4 °C, the secondary antibody for 1 h at room temperature and protein detected using the Clarity ECL substrate (Bio-Rad, #1705061) and the ChemiDoc Imaging System (Bio-Rad). Primary antibodies included Cell Signalling Technology antibodies p-AKT T308 1:1000 (#13038), AKT 1:1000 (#9272) and GAPDH 1:2000 (#5174), and BD Biosciences antibody RB 1:1000 (#554136). Secondary anti-rabbit-HRP (#7074) and anti-mouse-HRP (#7076) antibodies were sourced from Cell Signalling Technology. 

### 2.8. RNA Isolation and QRT-PCR

RNA from prostate cell lines (source: ATCC, mycoplasma free) was extracted using the Qiagen RNeasy mini kit (#74104), and all other RNA samples were isolated using the Qiagen AllPrep DNA/RNA/Protein Mini Kit (Qiagen, Manchester, UK #80004), according to the manufacturer’s instructions. RNA concentration and quality was confirmed using a NanoDrop 2000 (ThermoFisher, Loughborough, UK), and cDNA was synthesised using the Transcriptor 1st strand cDNA kit (Roche, Welwyn Garden City, UK, #04379012001) following the manufacturer’s instructions. QRT-PCR reactions were performed using 10 ng of cDNA and 2x qPCRBIO SyGreen Blue Mix (PCRBiosystems, London, UK, #PB20.16) following the manufacturer’s instructions in a QuantStudio 7 QRT-PCR machine with the following primers: *AR* (Exon 5-6): F 5′-CCTGGCTTCCGCAACTTACAC-3′, R 5′-GGACTTGTGCATGCGGTACTCA-3′, *AR-v7*: F 5′-CGTCTTCGGAAATGTTATGAAGC-3′, R 5′-GAATGAGGCAA-GTCAGCCTTTCT-3′ [41], *GAPDH*: F 5′-ACAGTTGCCATGTAGACC-3′, R 5′-TTGAGCACAGGGTACTTTA-3′) [42], *AXIN2*: F 5′-TCAAGACGGTGCTTACCTGT-3′, R 5′-TGCTGCTTCTTGATGCCATCA-3′ and *ASCL2*: F 5′-AAAGAACCCTTGACCTGGGG-3′, R 5′-AGATCTTGGCCAGCATGGA-3′. 

### 2.9. Ex Vivo Explant Preclinical Trials

Explants were performed as previously described [43] using a Leica V1200S vibratome to generate 250 µm slices from fresh tumour tissue embedded in 4% low melt agarose (Promega, Southampton, UK, #V21111) within 0.5–2 h post-dissection. Explants were cultured on 1 cm^3^ cubes of surgispon haemostat gelatine-based sponge (Vet Direct, Newcastle, UK, #SGSP001) treated with explant culture media (RPMI 1640 (ThermoFisher, #21875-059) supplemented with 10% foetal bovine serum (FBS, Sigma, Gillingham, UK, #F9665), penicillin/streptomycin (100 μg/mL; ThermoFisher, #15140122), gentamicin (0.1 mg/mL; Sigma, #G1272), amphotericin B (0.5 µg/mL, Fisher Scientific, Loughborough, UK, #15290-026), dihydrotestosterone (1 nM; Sigma, #D-073), hydrocortisone (0.01 mg/mL; Sigma, #H6909), insulin (0.01 mg/mL; Sigma, #I0516), ROCK inhibitor Y-27632 (5 µM; HelloBio, Bristol, UK, #HB2297)) and either docetaxel (10 nM; Stratech, Cambridge, UK, #A4394-APE), enzalutamide (10 µM; Stratech, #A3003-APE), olaparib (10 µM, Stratech, #S1060), capivasertib (1 µM; Stratech, #A1387-APE), or the DMSO control (Thermofisher, Loughborough, UK, #15303671). Explant tissues were treated for 48 h at 37 °C with atmospheric O_2_ and 5% CO_2_, fixed for 24 h at 4 °C in 10% neutral buffered formalin, and paraffin-embedded for IHC. Positive staining was quantified using semi-automated QuPath analysis software (latest v. 0.5.1) [44] (*n* = 3/treatment arm). 

### 2.10. In Vivo Preclinical Trials

Adult male (8 week-old) Athymic Nude mice (Charles River, Kent, UK) were bilaterally transplanted with tumour fragments subcutaneously and tumours were measured twice a week with callipers. Tumour volume was calculated using the modified ellipsoidal formula (V = ½ (Length × Width^2^). When tumours reached 100–300 mm^3^, mice were randomly assigned into treatment arms; docetaxel (10 mg/kg, i.p. once a week in 10% EtOH: 40% PEG 300: 5% Tween80: 45% saline), enzalutamide (10 mg/kg, p.o. daily 5 days a week in 10% DMSO: 40% PEG 300: 5% Tween80: 45% saline), or the equivalent dose of vehicle, using previously reported efficacious, non-toxic and well-tolerated drug doses [15].

### 2.11. Statistical Analysis

Statistical significance was determined using a one-way ANOVA with Tukey correction or an unpaired two-tailed *t* test (95% confidence interval) using GraphPad Prism 10 software, as indicated. A Monte Carlo simulation was employed to determine observed v expected enriched gene signature statistical significance in R (version 4.1.1) using 1,000,000 simulations to build a null distribution. *p* < 0.05 was considered statistically significant.

## 3. Results

### 3.1. Propagation of the CU-PC01 PDX Model

**Figure 1:**
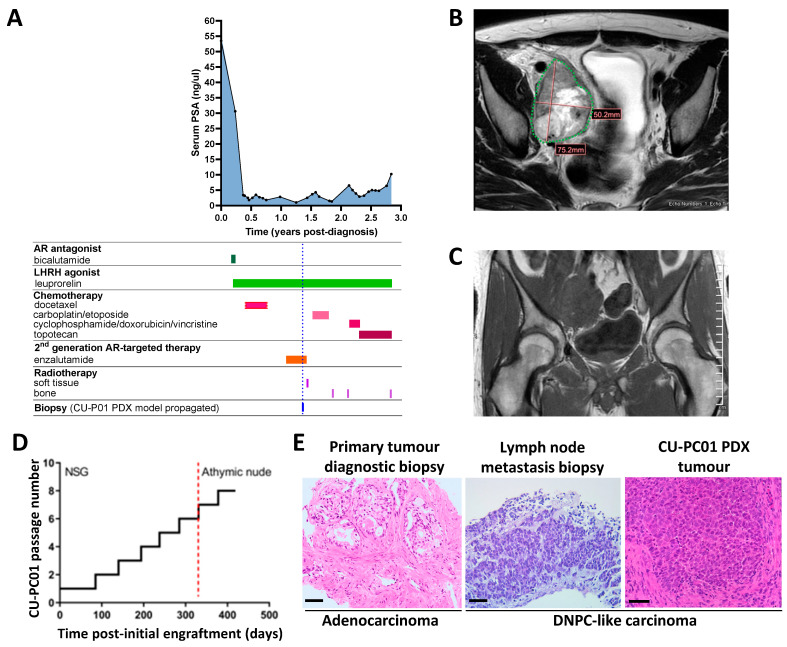
Generation of the CU-PC01 PDX model. (**A**) Graph displays the donor patient serum PSA levels over time from the point of diagnosis to death in relation to treatment (blue dashed line = lymph node image guided biopsy collection timepoint). (**B**) Axial T2 MRI and (**C**) coronal T1 MRI of the pelvis was performed to guide lymph node biopsy. (**D**) Plot displays the growth trajectory of the CU-PC01 PDX model from propagation to 419 days, involving six serial transplantations into adult male NSGs and two subsequent serial transplantations into adult athymic nude males (strain switch = red dashed line). The testosterone supplement was removed at the final passage (passage 8). (**E**) Representative H&E images of donor patient primary prostate adenocarcinoma at diagnosis (prostate carcinoma, left panel) and morphologically heterogeneous high-grade carcinoma with focal NE features in the lymph node metastasis biopsy (middle) and CU-PC01 PDX tumour (passage 1, right panel). Scale bar = 50 µm.

The CU-PC01 PDX model was established from a Caucasian man that presented with erectile dysfunction and a raised PSA (53.5 ng/mL) at 46 years old, with no family history of prostate cancer (although a second cousin had breast cancer). The patient was diagnosed with prostate adenocarcinoma (Gleason grade 4 + 4 = 8; ISUP grade group 4) and staging investigations revealed bulky, locally advanced disease with multiple pelvic and common iliac lymph node metastases and five bone metastases in the ribs, vertebrae, pelvis and proximal right femoral shaft, but no visceral metastases or distant lymphadenopathy (Stage T3b N1 M1b). The patient was commenced on conventional androgen deprivation therapy (ADT) in the form of an LHRH agonist (Prostap; leuprorelin) and a short course of an AR antagonist (bicalutamide) to cover for tumour flare. As his disease continued to progress, he was then treated with docetaxel chemotherapy, followed by the ARTT agent enzalutamide (Figure 1A). Although the patient’s PSA was reduced from 2.8 ng/mL to 1.0 ng/mL while on combined ADT and enzalutamide, increasing pelvic pain prompted repeat imaging which revealed an increase in the size of the right external iliac lymph node metastasis from 54.9 mm at baseline to 75.2 mm (maximal axis dimension; Figure 1B,C). This lymph node was then sampled via needle biopsy under radiological guidance, thereby providing tissue for PDX model development. No additional metastatic biopsies were collected from the donor patient.

Subcutaneous implantation of the lymph node metastasis biopsy into an immunocompromised adult male NSG mouse supplemented with testosterone revealed that CU-PC01 PDX tumours grow rapidly and approached ethical limits within 91 days (Figure 1D). Moreover, we observed CU-PC01 PDX tumours have a strong ability to be serially passaged in five subsequent transplantations into adult male NSG mice, which displayed an accelerated growth trajectory (ranging between 42–63 days). To determine if the CU-PC01 PDX tumours remain viable in mice with a more competent immune system, passage 7 was undertaken in an adult athymic nude male mouse that is deficient in mature T-cells, contrasting NSG mice that lack natural killer cells, mature B- and T-cells, and have defective macrophages and dendritic cells. Notably, CU-PC01 PDX tumour growth in the athymic nude mouse was comparable to the NSG mouse (Figure 1D, growth = 49 days). Moreover, the removal of the testosterone pellet at passage 8 did not hugely impact tumour growth rate compared to the previous passages (Figure 1D, growth = 42 days), indicative of androgen insensitive growth. Cryopreserved tumour fragments collected at passage 2 transplanted into NSG mice were also successfully propagated, and displayed a relatively similar growth rate (growth = 56 days, Supplementary Figure S1A). 

Histopathological analysis revealed that the high-grade prostate carcinoma in the patient lymph node metastasis biopsy displays heterogeneous cellular morphology with focal NE features, which are retained throughout serial transplantation and cryopreservation of the CU-PC01 PDX model. Remarkably, histopathology of the metastatic specimens contrast with the patient’s primary tumour specimen collected at diagnosis, which displayed prostate adenocarcinoma (Figure 1E and Supplementary Figure S1B–D). The lungs, liver, kidneys, bladder, prostate and lymph nodes were routinely collected from each subcutaneous CU-PC01 transplanted mouse and no overt metastases were observed at the time of dissection. This was further confirmed by H&E histological analysis of soft tissues and IHC to detect human mitochondria was negative, indicating that the CU-PC01 PDX model does not spontaneously metastasise to form secondary tumours (*n* = 11, 1 section per mouse, data available upon request). However, we cannot exclude the possibility that CU-PC01 disseminated cells or micrometastases arise but were undetected, and further soft tissues analysis, together with in vivo studies exploring non-subcutaneous implantation of AR-negative mCRPC CU-PC01 cells, is needed to determine the true metastatic potential of this model.

### 3.2. The CU-PC01 PDX Model Retains the Mutational Landscape of the Donor Patient

**Figure 2:**
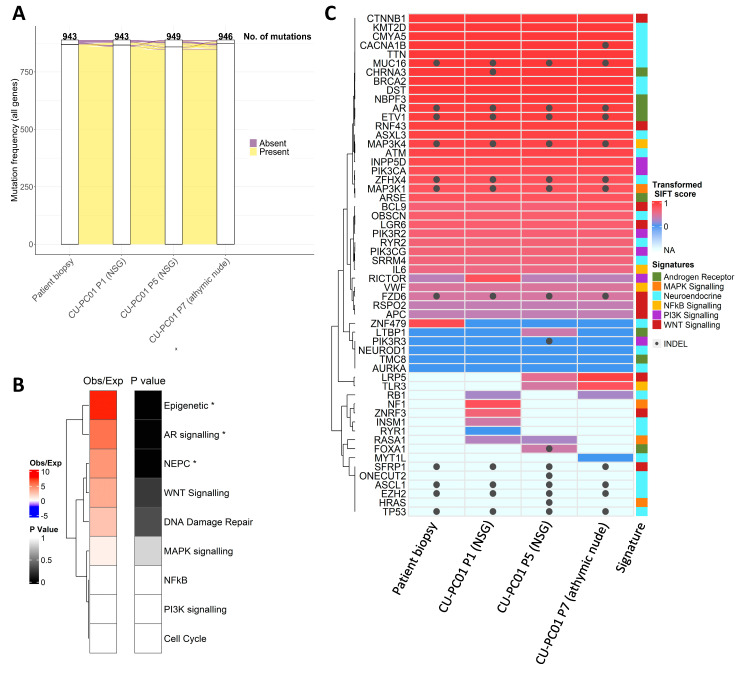
CU-PC01 PDX tumours genocopy the donor patient tumour with high concordance. (**A**) Alluvial plot generated using R ggalluvial package displaying the frequency of consensus SNV mutations that are present or absent in the patient lymph node biopsy and CU-PC01 PDX tumour specimens collected at P1, P5 and P7. A large majority of mutations are conserved in the CU-PC01 PDX tumours across all passages. (**B**) Enrichment of selected gene signatures in a consensus set of gene mutations present in all samples. The overlap of consensus genes with those in signatures is shown, as compared to the level expected by chance in 1,000,000 simulations. * indicates significant enrichment, defined as *p* < 0.05. (**C**) Heatmap shows genes from signalling pathways commonly deregulated in mCRPC (detailed in side bar), and genes with SNPs/INDELs present in the coding sequence (and UTRs) in the patient lymph node biopsy and/or CU-PC01 PDX tumour specimens collected at P1, P5, and P7. SIFT scores were transformed to 1 − x, thus a highly significant score is represented by a higher number. INDELs, Stop gain, and missense SNVs without a SIFT score are marked ‘NA’. Where multiple SIFT scores are present (e.g., multiple gene mutations detected), the most significant is used. INDELs = grey dot.

To determine if the mutational landscape of the CU-PC01 PDX model is preserved, relative to the donor patient, and retained during serial transplantation, whole exome sequencing (WES) was performed on DNA isolated from the patient biopsy and CU-PC01 PDX tumours at several passages. Analysis of the somatic mutational frequency of all genes revealed a high concordance between the patient lymph node metastatic biopsy and the CU-PC01 PDX tumours at passage 1 (P1) and passage 5 (P5) engrafted into NSG mice, and passage 7 (P7) engrafted into athymic nude mice (Figure 2A). Remarkably, 92–95% of mutated consensus genes within the patient lymph node biopsy were retained in the CU-PC01 PDX tumours (Supplementary Figure S2A), indicating that the mutational landscape of the patient lymph node biopsy is highly conserved in the CU-PC01 PDX model across multiple passages and in different immunocompromised mouse strains. All genetic variants identified by WES for each sample are detailed in full in Supplementary Table S2, where only a small number of genes were differentially altered between CU-PC01 PDX tumours at different passage numbers. Analysis of consensus genes present in all tumour samples analysed revealed significant enrichment of epigenetic, AR, and NEPC gene signatures, while WNT and DNA damage repair signalling displayed a trend for increased enrichment (Figure 2B). Noteworthy conserved deleterious mutations were detected in androgen receptor (*AR*), β-catenin (*CTNNB1*), ETS variant transcription factor 1 (*ETV1*), tumour protein p53 (*TP53*), lysine-specific histone methyltransferase lysine methyltransferase 2D (*KMT2D*), and breast cancer gene 2 (*BRCA2*) (Figure 2C, Table 1). Importantly, PCR analysis of genomic DNA confirmed the absence of spontaneous Epstein–Barr Virus (EBV) associated lymphoma (Supplementary Figure S2B), which has previously been reported in prostate cancer PDX generation [45]. 

The *AR* mutation identified at A646D (Figure 2C, Table 1 and Supplementary Table S2) lies within the hinge region of AR and is an SPOP binding consensus motif. Interestingly, the A646D mutation is reported to reduce AR:SPOP affinity and is frequently observed in patients with partial androgen insensitivity syndrome (PAIS), mild androgen insensitivity syndrome (MAIS), and prostate cancer [48,49] (COSMIC legacy identifier- COSM6906185 [50]). In addition, the conserved S33Y hotspot mutation in *CTNNB1* (β-catenin), which is known to activate the WNT pathway (residue S33 is a substrate for GSK3β phosphorylation [34]), is consistent with previous work indicating that deregulated WNT signalling contributes to NEPC/DNPC [51,52].

Several conserved *ETV1* missense mutations (G56A; G74A; G96A; G114A) and INDELs were also detected (Figure 2C, Table 1 and Supplementary Table S2). *ETV1* plays a key role in mediating androgen metabolism and is frequently over expressed in advanced prostate cancer, where chromosomal rearrangements (TMPRSS2 fusion) are common [53]. The multiple *ETV1* genetic alterations identified in the CU-PC01 PDX model are predicted to be pathogenic (Table 1, Supplementary Table S2). However, further functional genetic analysis is required to fully understand the consequence of these genetic variations. Of note, an *ETV1* mutation at G74 has been identified previously in a patient with metastatic prostate cancer [54].

NEPC and DNPC are commonly associated with RB (encoded by *RB1*) and p53 (encoded by *TP53*) loss [4,55,56,57,58,59], and WES analysis of both the lymph node patient biopsy and CU-PC01 PDX tumours revealed that genetic variants in *TP53* are conserved. These include several K > E missense variants associated with Li Fraumeni Syndrome ([60], entry #P04637) and a nonsense/stop gain mutation at residue 132 (E132X), a known oncogenic hotspot mutation within the DNA binding region that causes loss of function (Table 1) [61,62]. In addition, the *TP53* E171X stop gain mutation has also been observed previously in metastatic prostate cancer and is predicted to be tumorigenic [63]. Interestingly, only CU-PC01 PDX tumours at P1 and P7 carried an *RB1* missense mutation within the C-terminal domain (P793S) that is expected to be tolerated. Since loss of RB protein has been observed in NEPC without genetic alteration [57], we reasoned that RB could still be absent in the CU-PC01 model. To address this possibility, we performed IHC to detect RB in the patient biopsy and CU-PC01 tumours, revealing that RB is lost in the CU-PC01 PDX tumours (Supplementary Figure S3A). PC-3 xenograft tumours served as a positive control, as previously described [57], and IHC staining results were confirmed by Western Blotting (Supplementary Figure S3B,C).

NEPC has also been associated with deregulated DNA damage repair, including *BRCA2* loss [64]. We identified a conserved *BRCA2* missense mutation at A2595D in the CU-PC01 model that lies within the helical domain (Figure 2C, Table 1 and Supplementary Table S2), which is predicted to be deleterious (Table 1 and Supplementary Table S2). To our knowledge, the *BRCA2* A2595D mutation has not been detected in the clinic previously (none are reported in the COSMIC or cBioPortal databases [46,49]), although a missense *BRCA2* A2595G mutation has been identified previously in metastatic cutaneous squamous cell carcinoma [65]. These findings indicate that the CU-PC01 model may have impaired homologous recombination, however further work to assess the functional consequence of the *BRCA2* A2595D mutation is needed. Furthermore, a conserved E2678Q mutation in the tumour suppressor and epigenetic regulator *KMT2D* was also identified and is predicted to be deleterious, suggesting that the CU-PC01 model may have diminished methyltransferase activity.

### 3.3. The CU-PC01 PDX Model Expresses Both Basal and Luminal Prostate Epithelial Markers, Whereas NE Markers Are Absent or Weakly Expressed

**Figure 3:**
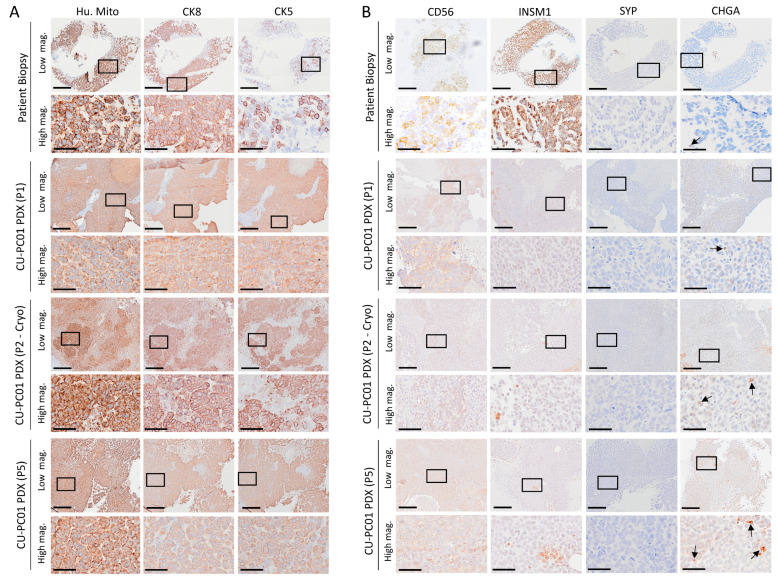
Characterisation of prostate epithelial cell populations within the CU-PC01 PDX model. Representative images of IHC staining of the patient lymph node metastatic biopsy and CU-PC01 PDX tumours collected at passage 1 (P1), a rederived cryopreserved tissue fragment at passage 2 (P2-Cryo) and at passage 5 (P5) to detect (**A**) human mitochondria (Hu. Mito), cytokeratin-8 (CK8) and cytokeratin-5 (CK5) staining and (**B**) NE markers CD56, INSM1, SYP and CHGA. Low magnification scale bar = 200 µm, high magnification scale bar = 50 µm (*n* = 3). Boxes indicate the high-magnification region.

To confirm that the CU-PC01 PDX cancer cells were of human origin, human mitochondria IHC staining was performed. As expected, human mitochondria positive staining was detected in the PDX tumours, similarly to the donor patient lymph node biopsy (Figure 3A). To characterise the prostate epithelial cell populations that reside within the CU-PC01 PDX model, IHC was performed to detect the basal and luminal prostate epithelial cell markers CK5 and CK8, respectively. CK5 and CK8 expression in PDX tumours (P1, P2-Cryo, and P5) were compared with the donor metastatic lymph node patient biopsy. Analysis of the luminal and basal epithelial cell markers revealed that the patient lymph node biopsy and CU-PC01 PDX tumours at various passages contain both CK5-positive and CK8-positive cells (Figure 3A). A high frequency of CK5 and CK8 expressing cells was observed, suggesting that the expansion of a transit amplifying (or “intermediate”) cell population of multipotential progenitors may have occurred [66], and is indicative of lineage plasticity [67]. 

To establish if the CU-PC01 PDX model expresses NE markers, IHC was performed to detect CD56, SYP, INSM1, and CHGA. IHC analysis revealed that SYP is absent in all samples, whereas weak, focal CD56 and INSM1 positive staining was detected in both the patient lymph node metastatic biopsy and CU-PC01 PDX tumours (Figure 3B). In addition, only rare CHGA positive cells were observed in the patient lymph node metastasis biopsy and the CU-PC01 PDX tumours (Figure 3B). The lack of apparent NE differentiation is supported by the absence of ASCL1, NEUROD1, and POU2F3, which are all transcription factors associated with small cell neuroendocrine carcinomas, including NEPC (Supplementary Figure S3D). Homeobox b13 (HOXB13), a core prostatic lineage pioneer factor that regulates prostate epithelial proliferation and differentiation, was also weakly expressed (Supplementary Figure S3D) [68]. Collectively, these findings indicate that CU-PC01 tumours do not display overt NE differentiation, raising the possibility that this model represents DNPC [4,55,69]. 

### 3.4. The CU-PC01 mCRPC PDX Model Is AR Negative

**Figure 4:**
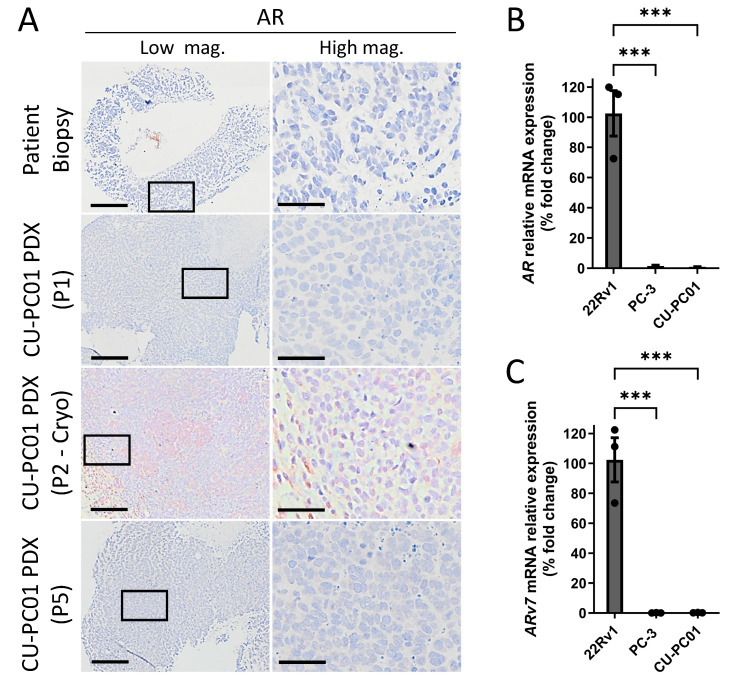
CU-PC01 mCRPC PDX tumours are AR-negative. (**A**) Representative IHC images for AR in the donor patient biopsy and CU-PC01 PDX tumours collected at P1, P2-cryo and P5 (n = 3). Low magnification scale bars = 200 µm, high magnification scale bars = 50 µm. Boxes indicate the high-magnification region. QRT-PCR analysis was performed on CU-PC01 PDX tumour specimens (P2) to detect (**B**) AR and (**C**) AR-v7 splice variant mRNA transcripts. 22Rv1 human CRPC cells = *AR* and *AR-v7* positive control. PC-3 human CRPC cells = *AR* and *AR-v7* negative control. Error bars = S.E.M (*n* = 3, 3 independent repeats). One-Way ANOVA with Tukey correction, *** *p* < 0.001.

Given that the donor patient progressed on ARTT (Figure 1A) and the presence of two conserved *AR* mutations, including the clinically relevant A646D mutation within the SPOP consensus motif (Table 1), we assessed if the AR protein is expressed in the CU-PC01 PDX model to better understand the molecular mechanism underpinning mCRPC growth and to ascertain if the model resembles DNPC. IHC to detect the N-terminus of AR revealed that AR is absent in the patient lymph node metastatic biopsy and CU-PC01 PDX tumours analysed at the early (P1) and late (P5) passage, as well as post-cryopreservation (P2-cyro) (Figure 4A). Furthermore, QRT-PCR to detect *AR* mRNA transcripts and the *AR-v7* splice variant revealed *AR* and *AR-v7* mRNA is significantly depleted in CU-PC01 PDX tumours relative to the positive controls (*n* = 3), consistent with DNPC (Figure 4B,C). Human mCRPC PC-3 cells served as a negative control, while 22Rv1 mCRPC cells provided a positive control [70]. Thus, AR is undetectable at the transcript and protein level, and this absence of AR is most likely the mechanism for ARTT resistance, rather than the missense mutations identified. In support, IHC to detect PSA and PSMA that are directly or indirectly regulated by AR, respectively, revealed that CU-PC01 PDX tumours are PSA and PSMA negative (Supplementary Figure S4A,B).

### 3.5. Characterisation of Wnt and PI3K Signalling Status in the CU-PC01 PDX Model

**Figure 5:**
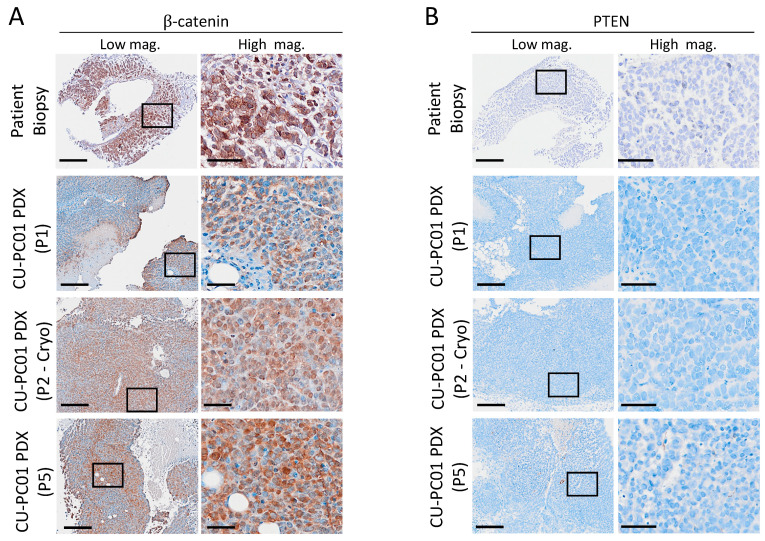
The CU-PC01 PDX model displays activated Wnt signalling and PTEN loss. Representative IHC images for (**A**) β-catenin and (**B**) PTEN in the donor lymph node metastatic patient biopsy and CU-PC01 PDX tumours collected at P1, P2-cryo, and P5 (*n* = 3) show a high level of nuclear β-catenin and the absence of PTEN, respectively. Low magnification scale bars = 200 µm, high magnification scale bars = 50 µm. Boxes indicate the high-magnification region.

Both the Wnt and PI3K signalling pathways are commonly deregulated in prostate cancer and can contribute to disease progression and drug resistance [31,34,71,72]. Given that several genetic alterations in the Wnt and PI3K pathways were conserved in the CU-PC01 PDX model (Figure 2C, Table 1, Supplementary Table S2), key components of these cascades were analysed to determine the status of Wnt and PI3K pathway activity. IHC for the intracellular component of the Wnt pathway *β*-catenin was performed, revealing that nuclear *β*-catenin is present in both the patient lymph node biopsy and the CU-PC01 PDX tumours (Figure 5A). This finding is likely to be attributable to the observed S33F *CTNNB1* gain of function mutation known to constitutively activate *β*-catenin [34], and correlates with the upregulated transcription of canonical Wnt target genes *ASCL2* and *AXIN2* relative to control cell lines that do not express Wnt pathway driver mutations (namely, non-malignant human prostate RWPE-1 cells and mCRPC PC-3 and 22Rv1 cell lines) (Supplementary Figure S4C,D). Similarly to *APC* mutant colon tumours, we do not observe nuclear β-catenin throughout the CU-PC01 tumours, indicating that the additional deregulation of the Wnt pathway from the tumour microenvironment is also involved in activating the pathway even in *CTNNB1* mutant cells [73]. Furthermore, the presence of mutations in several other components of the Wnt pathway (Table 1 and Supplementary Table S2), including deleterious mutations in the E3 ubiquitin ligase *RNF43* that regulates Wnt receptor turnover [74], may also play a role in mediating the level of Wnt pathway activity. 

WES analysis revealed a number of conserved genetic alterations within the PI3K pathway, however they were all predicted to be tolerated (Table 1). Synonymous mutations with no known functional impact were also observed in the tumour suppressor phosphatase and tensin homolog (*PTEN*) that negatively regulates the PI3K cascade (Supplementary Table S2). These findings indicate that the CU-PC01 PDX model does not carry a PI3K pathway driver mutation, yet IHC staining for the C-terminus of PTEN revealed the CU-PC01 model is negative for PTEN protein (Figure 5B). This finding was supported by IHC with another PTEN antibody (Neomarkers, #10P03, data available upon request). In corroboration with PTEN loss, activation of AKT signalling in CU-PC01 PDX tumours was confirmed by Western Blotting for p-AKT (T308) and IHC to detect AKT downstream substrates p-4EBP1 (T37/46) and p-RPS6 (S235/236) in CU-PC01 tumours (Supplementary Figure S3). Taken together, these data show that CU-PC01 tumours are associated with oncogenic Wnt and PI3K signalling, presenting direct mechanisms for DNPC-like growth and ARTT resistance [31,75]. 

While *PTEN* homozygous deletion is currently predicted to occur in 29% of DNPC cases [4], the frequency of PTEN loss at the protein level remains unknown. Since *PTEN* genetic alterations were not detected in CU-PC01 tumours (Supplementary Table S2), future work to determine the cause of PTEN loss in this model is needed. It is possible that PTEN post-translational modifications or epigenetic silencing may cause PTEN loss. Several phosphorylation sites within the C-tail of domain of PTEN have previously been shown to reduce PTEN protein levels, stability and phosphatase activity [76,77], and although rare, epigenetic silencing of PTEN has also been identified in prostate cancer previously [75,78].

### 3.6. CU-PC01 PDX Tumours Are Resistant to Docetaxel and Enzalutamide Monotherapy

**Figure 6:**
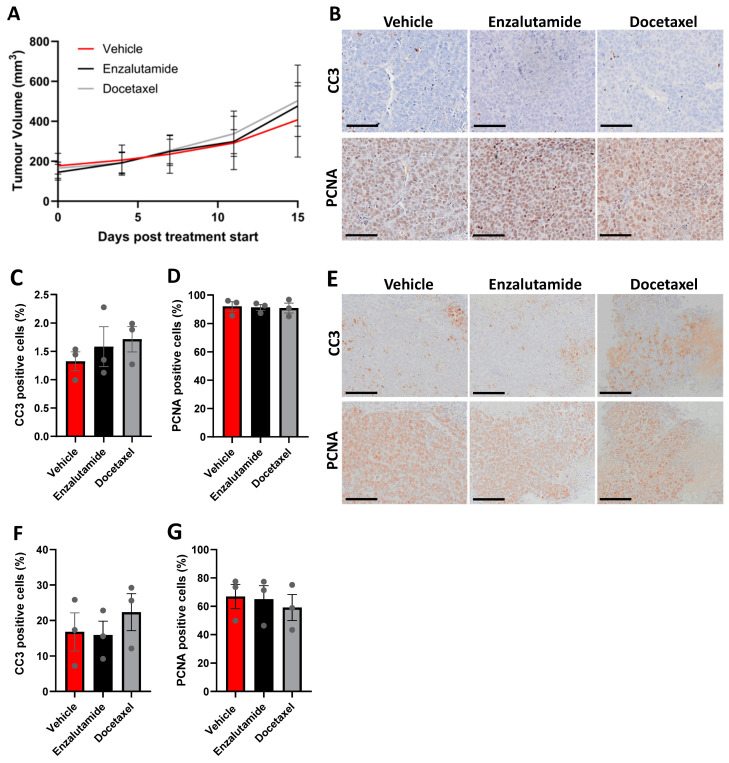
CU-PC01 PDX tumours are resistant to enzalutamide and docetaxel. (**A**) Chart displays tumour growth for subcutaneous CU-PC01 PDX tumours in adult male athymic nude mice treated with either vehicle, enzalutamide (10 mg/kg by gavage 5 days on 2 days off), or docetaxel (10 mg/kg i.p. once a week) for 15 days. Error bars represent S.E.M, Two-way ANOVA = not significant, *p* = 0.9748 (*n* = 4 or 6/treatment arm). (**B**) Representative IHC images and (**C**,**D**) quantitation for the apoptosis marker cleaved-caspase-3 (CC3) and proliferation marker PCNA in CU-PC01 PDX tumours treated with either enzalutamide or docetaxel in vivo for 15 days (*n* = 3/treatment arm). No significant difference was observed between the treatment arms (one-way ANOVA with Tukey correction, *p* ≥ 0.5683). (**E**) Representative IHC images and (**F**,**G**) quantitation for CC3 and PCNA CU-PC01 PDX tumour explant cultures treated with either enzalutamide or docetaxel for 48 h ex vivo (*n* = 3/treatment arm). Error bars represent S.E.M. No significant difference was observed between the treatment arms (one-way ANOVA with Tukey correction, *p* ≥ 0.6390). Scale bars = 100 µm.

Treatment options for advanced CRPC are limited owing to the emergence of drug resistant clones that are insensitive to current treatment options and survival outcome remains poor [79,80]. To test the efficacy of two standard treatments for mCPRC, enzalutamide and docetaxel, we performed in vivo preclinical trials using the CU-PC01 PDX model. Cohorts of adult athymic nude males were treated with either docetaxel, enzalutamide or vehicle control (*n* = 4–6/treatment arm) for 15 days. No statistical significant difference in tumour growth was observed in response to enzalutamide or doxetaxel treatment relative to vehicle treated control mice (Figure 6A), indicating that the CU-PC01 model is resistant to enzalutamide and docetaxel treatment. 

In support, endpoint tumour weights were also comparable between the treatment arms (Supplementary Figure S5) and quantitation of IHC to detect the apoptotic marker Cleaved caspase-3 (CC3) and the proliferation marker PCNA revealed that the number of PCNA and CC3 positive cells is unaltered in CU-PC01 tumours in response to enzalutamide or docetaxel treatment (Figure 6B–D). 

To establish if CU-PC01 PDX explant cultures provide a valuable means for rapid, short-term preclinical studies exploring new therapies to treat AR-negative mCRPC and/or sensitize them to enzalutamide or docetaxel, CU-PC01 PDX tumours were harvested when approaching ethical limits, and ex vivo explant cultures established. We show that CU-PC01 explants are resistant to both docetaxel and enzalutamide treatment for 48 *h* and that the number of apoptotic and proliferative cells remain unchanged (Figure 6E–G), correlating with the in vivo findings. Together, these findings indicate that the CU-PC01 PDX model is resistant to enzalutamide and docetaxel, mirroring the donor patient’s response to these agents in the clinic (Figure 1A) and validate the utility of ex vivo assays for preclinical studies in this setting.

### 3.7. CU-PC01 PDX Ex Vivo Explants Are Sensitive to the PARP Inhibitor Olaparib

**Figure 7:**
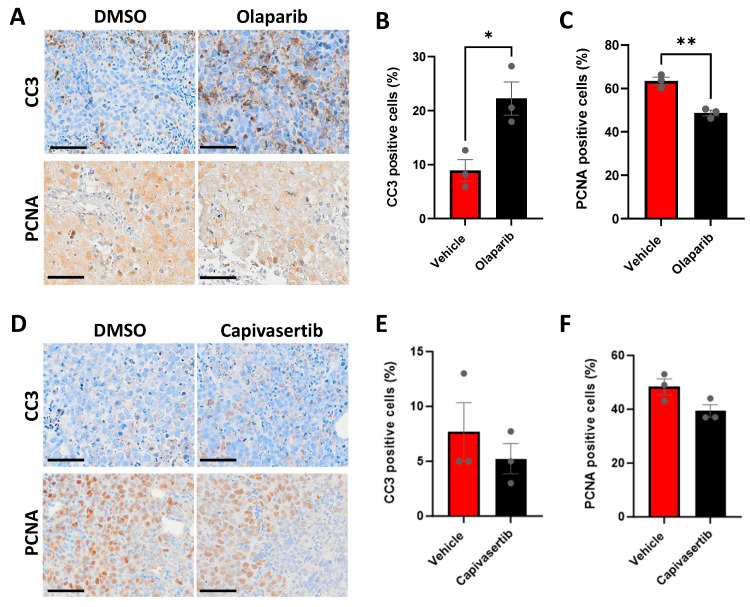
CU-PC01 PDX ex vivo explants are sensitive to PARP inhibition. (**A**) Representative IHC images for CC3 and PCNA, and quantitation of the percentage of (**B**) CC3 and (**C**) PCNA positive cells in CU-PC01 PDX tumour explant cultures treated with either vehicle (DMSO) or olaparib (10 µM) for 48 h (*n* = 3 independent repeats, in triplicate). Olaparib significantly increased the percentage of CC3-positive cells and significantly reduced the percentage of PCNA-positive cells (unpaired two-tailed t test; * *p* < 0.05, ** *p* < 0.01). (**D**) Representative IHC images for CC3 and PCNA, and quantitation of the percentage of (**E**) CC3 and (**F**) PCNA positive cells in CU-PC01 PDX tumour explant cultures treated with either vehicle (DMSO) or capivasertib (1 µM) for 48 h (*n* = 3 independent repeats, in triplicate). No significance was observed (unpaired two-tailed t test, *p* ≥ 0.0731). Error bars = S.E.M.

Having established a rapid ex vivo PDX explant preclinical testing platform for the CU-PC01 model, the short-term efficacy of the poly (ADP-ribose) polymerase (PARP) inhibitor olaparib was explored to ascertain if PARP inhibition is efficacious against CU-PC01 PDX tumours carrying a *BRCA2* mutation with unknown pathogenicity. Interestingly, a significant increase in the percentage of CC3-positive tumour cells was observed following olaparib treatment, which was accompanied by a significant decrease in the percentage of PCNA-positive tumour cells (Figure 7A–C), suggesting the CU-PC01 PDX model is sensitive to PARP inhibition ex vivo.

To better understand the requirement for PI3K/AKT signalling in the PTEN-deficient CU-PC01 PDX model, we also treated CU-PC01 explants with the AKT inhibitor capivasertib (AZD5363). While AKT inhibition did not alter the number of CC3-positive apoptotic cells relative to the vehicle control, a marginal trend for a reduction in the percentage of PCNA-positive cells was observed in CU-PC01 PDX explants upon capivasertib treatment (Figure 7D–F), albeit not statistically significant. Thus, the CU-PC01 model appears to lacks initial sensitivity to AKT inhibition in this ex vivo setting. However, it will be important for future work to investigate AKT inhibitor efficacy in this model upon long-term treatment with/without ARTT in vivo, which is reported to be beneficial in PTEN-negative mCRPC patients [81,82].

## 4. Discussion

AR-negative prostate cancer is a highly aggressive, incurable form of prostate cancer that occurs in over a third of mCRPC patients and rarely develops in the absence of ARTT [4,83]. To improve our clinical management of AR-negative mCRPC, new therapies and molecular biomarkers are needed, which hinge on the development and study of a wide range of prostate cancer models that recapitulate the broad spectrum of clinicopathological features associated with all mCRPC variants, including AR-negative adenocarcinoma, NEPC, DNPC and mixed/heterogeneous morphologies. Accordingly, we have developed an entirely new model of advanced AR-negative mCRPC that phenocopies the original patient biopsy with high concordance in terms of histopathology, genomic landscape, DNPC-like features and enzalutamide/docetaxel resistance. 

Significantly, the CU-PC01 PDX model exhibits several molecular characteristics of AR-negative mCRPC commonly observed in mCRPC variants such as NEPC and DNPC (e.g., AR, PSA and PSMA negative, PTEN and RB loss and genetic aberrations including a *TP53* stop gain mutation and a *CTNNB1* activating mutation [4,35,46,47,55,56,57,58,64,84,85,86,87,88,89,90,91,92] (summarized in Table 2). However, the CU-P01 PDX model lacks expression of key NE markers (e.g., SYP, ASCL1, NEUROD1 and POU2F3), and only exhibits weak/rare focal expression of INSM1, CD56, and CHGA. Collectively, these data indicate that the AR-negative mCRPC CU-PC01 PDX classifies as a model consistent with DNPC, representing a valuable, clinically relevant tool for testing new therapeutic avenues to treat this incurable disease.

Although clinical data describing the prevalence of protein and genetic perturbations in distinct mCRPC variants is beginning to emerge in the literature, current evidence indicates *TP53* mutation and the loss of RB and PTEN are among the most frequently altered molecular aberrations in DNPC, NEPC and adenocarcinoma mCRPC subtypes; *TP53* mutation incidence = 57%, 37–67% and 31–40%, respectively, *RB1* CNV/mutation/protein loss incidence = 29%, 37–90% and 16%, respectively, and *PTEN* mutation/CNV/protein loss incidence = 29%, 37–63%, 32%, respectively [4,35,54,55,57]. Indeed, 94% (17/18) of the genes with a conserved deleterious/damaging mutation identified in the CU-PC01 PDX model (Table 1) have been observed previously in patients with NEPC (Supplementary Table S4) [35,46,47,55,84] and 100% of mCRPC adenocarcinomas [54,93,94,95]. These findings further emphasize that some common features exist between different mCRPC subtypes, presenting a key challenge for their classification.

As the treatment landscape evolves and the range and frequency of diverse mCRPC subtypes increases, the demand for new classification strategies for mCRPC to aid treatment decisions and clinical trial design is mounting. While the classification of DNPC is currently AR and NE marker-negative, this approach may prove to be a rather broad classification system that encompasses multiple mCRPC disease subtypes with unique genetic, histopathological, metabolic and biochemical manifestations, and new markers to distinguish mCRPC variants are needed. Indeed, the weak and/or rare focal expression of CD56, INSM1, and CHGA in the AR-negative mCRPC CU-PC01 model that lacks classic NE markers (SYP, ASCL1, NEUROD1, and POU2F3) illustrates that unique morphologies exist that remain to be fully classified. Moreover, the variance in NE marker expression within different mCRPC subtypes indicates that a diverse set of events may underpin their formation and progression. Interestingly, the high prevalence of both epithelial cell markers (CK5 and CK8) and the weak focal expression of HOXB13, CD56 and INSM1 in the CU-PC01 model supports the concept of epithelial transdifferentiation with lineage plasticity upon ARTT [55,96,97,98]. However, the genetic analysis of the primary tumour at diagnosis is needed to determine if DNPC-like features emerged through divergent clonal evolution and, unfortunately, attempts to isolate DNA from the diagnostic block were unsuccessful owing to poor quality DNA and low yield.

Ultimately, further investigations are required to (i) determine the true degree of mCRPC subtypes, (ii) increase our understanding of the significance of unique NE profiles and (iii) to identify new biomarkers to classify them. Collectively, these studies could fundamentally advance our understanding and management of this lethal disease. Determining the prevalence and predictive value of epithelial and NE markers in mCRPC subtypes could also provide valuable insights into how distinct mCRPC variants arise, and aid the development of novel subclassification systems for mCRPC that can inform clinical decisions. In the advent of cutting-edge single-cell technologies such as digital spatial profiling, it will be interesting to discover if the abundance, spatial distribution and/or heterogeneity of certain NE and/or prostate basal/luminal epithelial cell markers during the evolution of distinct mCPRC variants correlates with key clinicopathological features, overall outcome and/or treatment response.

Currently, AR-negative mCRPC clinical outcomes remain poor and new therapies are urgently needed. Our ex vivo and in vivo CU-PC01 PDX preclinical studies corroborate clinical findings that AR-negative mCRPC tumours are resistant to ARTT (enzalutamide) and chemotherapy (docetaxel) [2,8,83] mirroring the donor patient, and strengthen the rationale to explore PARP inhibitors in AR-negative mCRPC variants with a *BRCA2* mutation. PARP inhibitors have recently been granted FDA approval for the treatment of mCRPC with a DNA damage repair mutation (e.g., *BRCA1/2*), having shown improved clinical outcomes in a series of clinical trials, however efficacy against AR-negative mCRPC (with or without NE markers) is currently not reported [99,100,101,102,103,104]. Clinical trials exploring AKT small molecule inhibitors in the clinic have also recently begun to show promise in mCRPC patients in combination with chemotherapy (docetaxel) [105], or together with ARTT (abiraterone) in PTEN-deficient mCPRC patients [82]. Nevertheless, it is not yet known if AKT inhibitors are efficacious against AR-negative mCRPC. The CU-PC01 PDX model provides a new tool to investigate the functional importance of PTEN loss in AR-negative mCRPC, and to explore PI3K/AKT-pathway targeted therapies. Although our short-term ex vivo explant experiment revealed that CU-PC01 tumours are not initially sensitive to AKT inhibition, future work to explore the long-term efficacy of PI3K/AKT-directed therapy in vivo is warranted to inform AR-negative mCRPC treatment decisions in the clinic.

The constitutive activation of *β*-catenin also presents an actionable target for AR-negative mCRPC modelled by the CU-PC01 PDX, and *CTNNB1* mutations have been observed in 4.3–5.4% of mCRPC cases (with unknown AR/NE status) [34]. Although Wnt pathway targeted therapies have not been directly explored in patients with mCRPC, findings from a Phase I clinical trial exploring the porcupine inhibitor LGK974 (that blocks Wnt ligand secretion) in untreatable solid cancers may provide valuable proof-of-concept data [34,106]. It will be interesting for future studies to explore if the blockade of the Wnt/*β*-catenin pathway can suppress CU-PC01 tumour growth in vivo. Furthermore, the CU-PC01 model provides a valuable resource to advance our molecular understanding of the complex interactions between the PI3K/PTEN/AKT and Wnt pathways during mCRPC, owing to the concomitant loss of PTEN and constitutive activation of *β*-catenin [31,75,77,107].

In summary, continuing to build a collection of well-characterized mCPRC models spanning diverse subtypes of this disease is central to progressing our molecular understanding of mCRPC variants, aiding their subclassification and accelerating the discovery of novel therapeutic strategies and predictive biomarkers that can improve mCRPC outcomes.

## Figures and Tables

**Table 1:** Summary of CU-PC01 PDX model conserved genetic aberrations.

Gene	Mutation Type	Genetic Alteration ^1^	Allelic Status	Predicted Impact ^2^
*AR signalling*				
*AR*	Missense INDEL	A646D; A114D Non-Fs: p.457_469del/p.457_469del	Hom Hom	Deleterious NR
*ARSE*	Missense	G379S; G424S; G449S	Hom	Tolerated
*CHRNA3*	Missense	R37H	Hom	Deleterious
*ETV1*	Missense INDEL	G56A; G74A; G96A; G114A UTR5: c.-31delT	Het Hom	Deleterious NR
*LTBP1*	Missense	V957A; V999A; V1010A;V1052A; V1378A	Hom	Tolerated
*NBPF3*	Missense	Y114C; Y58C	Hom	Deleterious
*TMC8*	Missense	N306I	Hom	Tolerated
*NF-κB signalling*				
*MAP3K1*	Missense INDEL	D806N Non-Fs: p.941_942del	Hom Hom	Damaging Benign
*MAP3K4*	Missense	H906P; H359P	Het	Tolerated
	INDEL	Non-Fs: p.P27delinsPP/p.1185_1186del	Hom	NR
*IL6*	Missense	D86E; D162E	Het	Tolerated
*VWF*	Missense	H484R; Q852R; T1381A; F2561Y*	Hom	Tolerated
*Epigenetic regulators*				
*KMT2D*	Missense	E2678Q	Het	Deleterious
*NEPC-associated genes*				
*TP53*	Stop Gain INDEL	E12X*; E39X*; E132X*; E171X* UTR5 c.-179_-181delAAA	Hom Hom	LOF NR
*TTN*	Missense	R25794Q; R25919Q; R25986Q; R32291Q; R33218Q; R34859Q	Het Het	Deleterious Deleterious
*DST*	Missense	P1984H; P2310H; P2350H; P2488H	Het	Deleterious
*MUC16*	Missense INDEL	S3337L; V3530I; T3788I; G3826E; H4166N; I4902V; V9909I; E12290K; T10155I S7019L; N13438D Fs: p.P13560fs/p.K13558fs	Hom Hom Het Het	Deleterious Deleterious Deleterious NR
*ZFHX4*	Missense INDEL	P1273S UTR3: c.*48delA	Het Het	Possibly damaging NR
*ZNF479*	Missense	Y135C; M369T	Het	Tolerated
*CACNA1B*	Missense	L2215R	Hom	Deleterious
*CMYA5*	Missense	L1669S	Hom	Deleterious
*OBSCN*	Missense	A7172V; A8129V	Hom	Tolerated
*RYR2*	Missense	G1886S	Het	Tolerated
*AURKA*	Missense	I57V	Het	Tolerated
*ASXL3*	Missense	N954S	Hom	Tolerated
*NEUROD1*	Missense	T45A	Hom	Tolerated
*SRRM4*	Missense	S243N	Het	Tolerated
*ASCL1*	INDEL	Non-Fs: p.A50delinsAQ	Het	Benign
*EZH2*	INDEL	UTR3: c.*21delC	Hom	Benign
*DNA damage repair*				
*ATM*	Missense	Y1475C*; D1853N*	Hom	Possibly damaging
*BRCA2*	Missense	A2595D*	Hom	Deleterious
*PI3K signalling*				
*INPP5D*	Missense	H1169Y; H1168Y	Het	Tolerated
*PIK3CA*	Missense	I391M	Het	Tolerated
*PIK3CG*	Missense	M35K; T857A	Het	Tolerated
*PIK3R2*	Missense	S234R; S313P	Hom	Tolerated
*PIK3R3*	Missense	N127K; N202K; N329K; N239K; N283K	Het	Tolerated
*RICTOR*	Missense	S837F; S552F	Het	Tolerated
*Wnt signalling*				
*FZD6*	Missense	M40L; M345L; M313L	Hom	Tolerated
	INDEL	UTR3: c.*202delA	Hom	NR
*LGR6*	Missense	V453A; V592A; V540A; V636M; V775M; V723M	Het Het	Tolerated Damaging
*RSPO2*	Missense	L122P; L119P; L186P	Hom	Tolerated
*SFRP1*	INDEL	Non-Fs: p.13_14del	Het	NR
*APC*	Missense	V1804D; V1822D	Hom	Tolerated
*BCL9*	Missense	P332L	Het	Tolerated
*CTNNB1*	Missense	S33Y; S26Y	Het	Deleterious (GOF)
*RNF43*	Missense	I47V P104L; P231L L291M; L418M	Het Het Het	Tolerated Deleterious Possibly damaging

^1^ Mutations observed previously in clinical prostate cancer specimens are underlined (source: all prostate cancer genomic datasets available in cBioPortal [46,47] (cBioportal.org accessed December 2023)). Conserved stop gain mutations, INDELs, or a mutation with a SIFT score are shown (i.e., present in the donor patient lymph node biopsy and CU-PC01 PDX tumours collected at P1, P5 and P7, in accordance with Figure 2C). Fs = frameshift. X = a nonsense/stop gain mutation. Mutations with an asterix (*) indicate a homozygous mutation in CU-PC01 tumours and a heterozygous mutation in the patient biopsy. Allelic status data for all genes is provided in Supplementary Table S3. Het = heterozygous, Hom = homozygous. ^2^ Prediction impact of SNV mutation according to the SIFT score, where <0.05 = deleterious (i.e., pathogenic), and 0.05–1.0 = tolerated (benign) or PolyPhen2_HDIV score if no SIFT score is available; 0–0.452 = benign, 0.453–0.956 = possibly damaging, 0.957–1.0 = damaging. LOF = loss of function, GOF = gain of function. INDEL clinical significance (CLNSIG) results are shown where available. NR = Not reported.

**Table 2:** Summary of key molecular perturbations in the CU-PC01 PDX model.

Protein Alterations		Genetic Alterations	
Molecular Marker	CU-PC01 PDX Status	Genetic Variant	CU-PC01 PDX Status
*Cell differentiation*		*Known driver mutation*	
CK5	Positive	*CTNNB1* (β-catenin)	Activating mutation (GOF)
CK8	Positive	*TP53*	Stop gain mutation (LOF)
AR	Negative	*BRCA2*	Missense mutation (LOF?)
SYP	Negative		
CHGA	Rare	*Candidate driver mutation*	
CD56	Focally weak	*DST*	Missense mutation
INSM1	Focally weak	*KMT2D*	Missense mutation
ASCL1	Negative	*MUC16*	Missense mutation
NEUROD1	Negative	*TTN*	Missense mutation
HOXB13	Weak	*ZFHX4*	Missense mutation
*Tumour suppressor*			
PTEN	Negative		
RB	Negative		
*Oncogene*			
Nuclear β-catenin	Positive		

## Data Availability

Whole exome sequencing datasets are available in the NCBI SRA database (https://www.ncbi.nlm.nih.gov/sra/PRJNA1086884, created March 2024) under the accession number PRJNA1086884. Additional data generated in this study are available from the corresponding author on reasonable request. The CU-PC01 PDX model is available under a material transfer agreement with Cardiff University.

## Supplementary Materials

The following supporting information can be downloaded at: https://www.mdpi.com/article/10.3390/cells13080673/s1, Figure S1: Histopathological analysis of CU-PC01 PDX tumours revealed phenotypic preservation across serial passages and post-cryopreservation; Figure S2: The mutational landscape of the donor patient biopsy is well-conserved in the CU-PC01 PDX tumour model across multiple passages; Figure S3: CU-PC01 PDX tumours display RB loss, weak HOXB13 expression and lack several NE markers; Figure S4: The CU-PC01 PDX model does not express PSA or PSMA and displays elevated Wnt and PI3K signalling; Figure S5: Enzalutamide and docetaxel treatment has no effect on CU-PC01 PDX tumour burden; Table S1: Gene list of signalling pathways and cellular processes that are commonly altered in prostate cancer; Table S2: CU-P01 PDX model whole exome sequencing data summary; Table S3: CU-P01 PDX model allelic frequency data summary; Table S4: Frequency of genetic alterations in NEPC patient specimens for all conserved genetic variants detected in CU-PC01 PDX tumours.
