# Supplementary material for: Development and Characterisation of a New Patient-Derived Xenograft Model of AR-Negative Metastatic Castration-Resistant Prostate Cancer
Source: Cells. 2024 Apr 12;13(8):673. doi: 10.3390/cells13080673 (PMC11049137; doi:10.3390/cells13080673)
Supplement: Supplementary file 1 [file cells-13-00673-s001.zip › Figure S1-revision.pptx]

## Slide 1
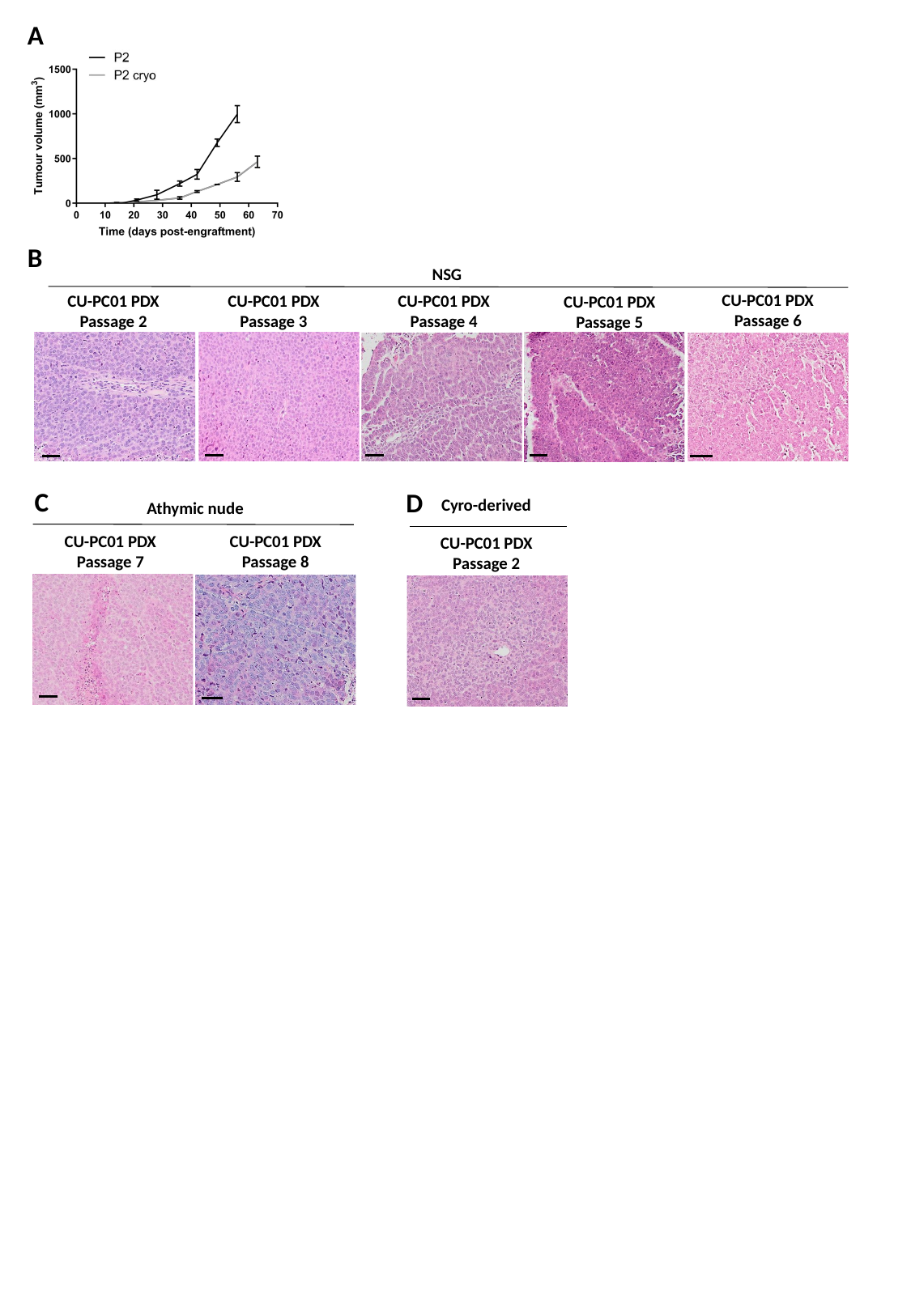

A
B
NSG
CU-PC01 PDX
Passage 6
CU-PC01 PDX
Passage 2
CU-PC01 PDX
Passage 3
CU-PC01 PDX
Passage 4
CU-PC01 PDX
Passage 5
C
D
Cyro-derived
Athymic nude
CU-PC01 PDX
Passage 7
CU-PC01 PDX
Passage 8
CU-PC01 PDX
Passage 2
