# Supplementary material for: Development and Characterisation of a New Patient-Derived Xenograft Model of AR-Negative Metastatic Castration-Resistant Prostate Cancer
Source: Cells. 2024 Apr 12;13(8):673. doi: 10.3390/cells13080673 (PMC11049137; doi:10.3390/cells13080673)
Supplement: Supplementary file 1 [file cells-13-00673-s001.zip › Figure S2 - revision.pptx]

## Slide 1
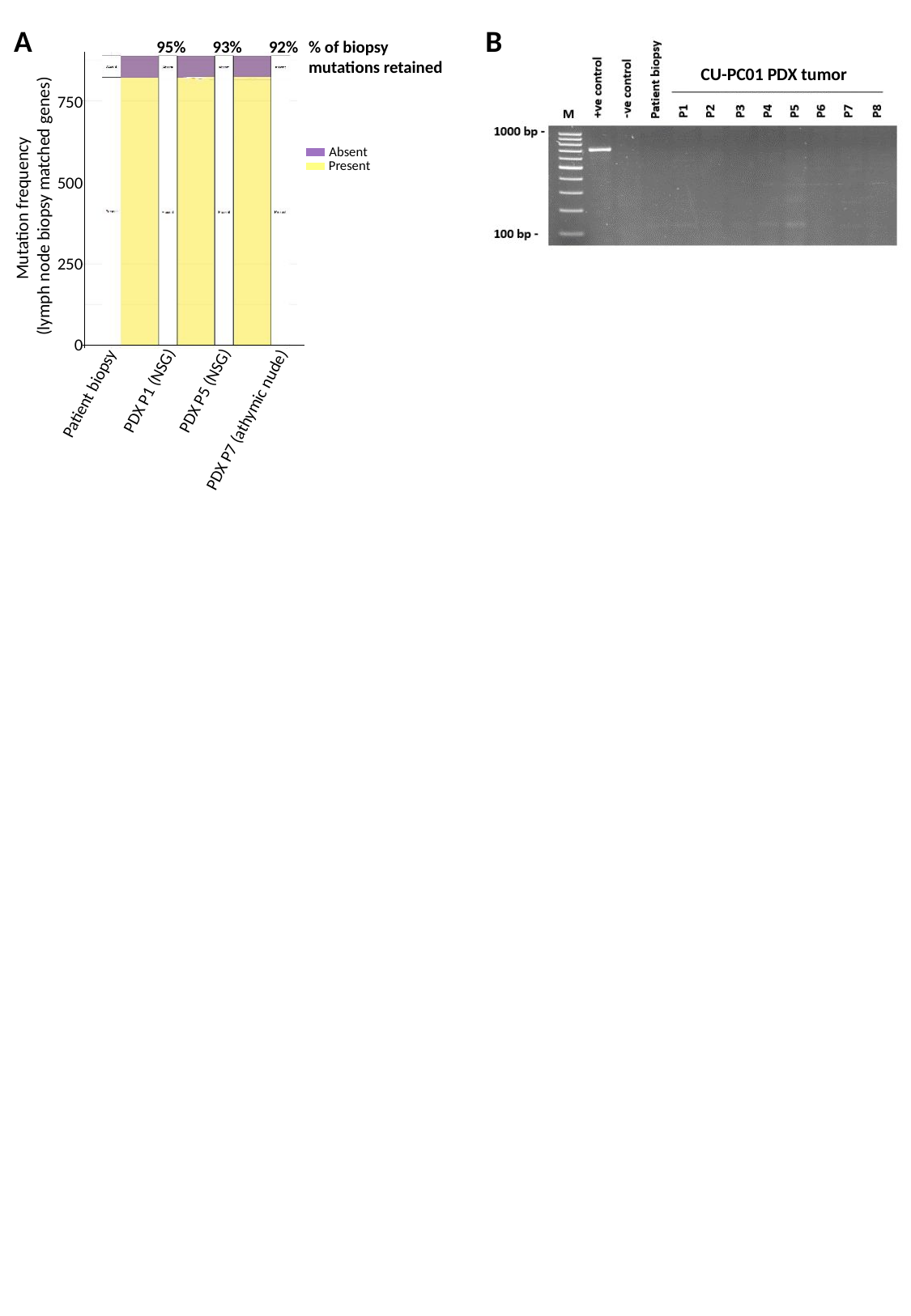

A
B
95% 93% 92%
% of biopsy mutations retained
CU-PC01 PDX tumor
750
500
250
0
Absent
Present
Mutation frequency
(lymph node biopsy matched genes)
PDX P1 (NSG)
PDX P5 (NSG)
Patient biopsy
PDX P7 (athymic nude)
