# Supplementary material for: Development and Characterisation of a New Patient-Derived Xenograft Model of AR-Negative Metastatic Castration-Resistant Prostate Cancer
Source: Cells. 2024 Apr 12;13(8):673. doi: 10.3390/cells13080673 (PMC11049137; doi:10.3390/cells13080673)
Supplement: Supplementary file 1 [file cells-13-00673-s001.zip › Figure S3 - revision.pptx]

## Slide 1
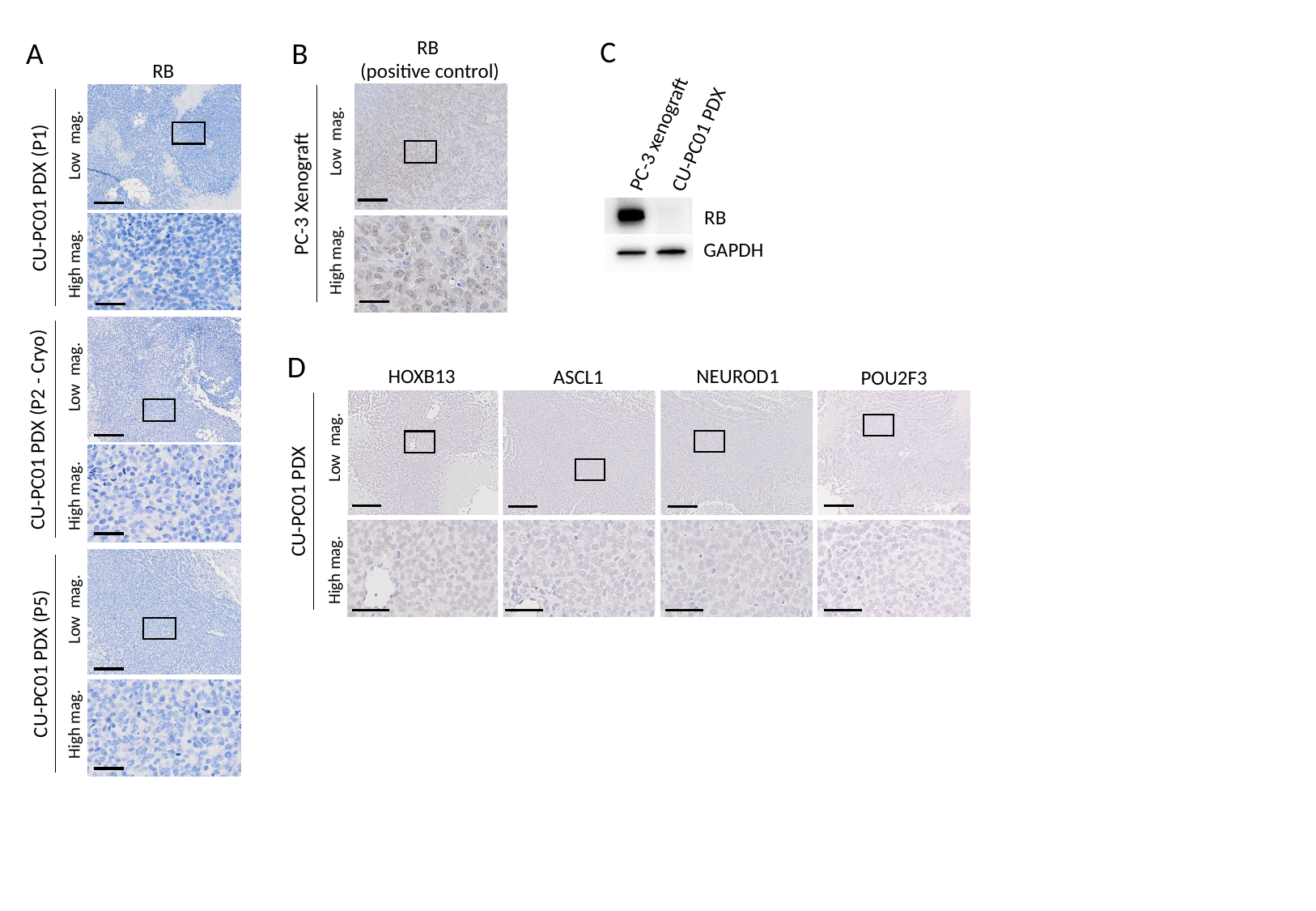

C
RB
(positive control)
B
A
PC-3 xenograft
CU-PC01 PDX
RB
GAPDH
RB
High mag. Low mag.
High mag. Low mag.
PC-3 Xenograft
CU-PC01 PDX (P1)
D
HOXB13
NEUROD1
ASCL1
POU2F3
High mag. Low mag.
CU-PC01 PDX (P2 - Cryo)
High mag. Low mag.
CU-PC01 PDX
High mag. Low mag.
CU-PC01 PDX (P5)
