# Supplementary material for: Development and Characterisation of a New Patient-Derived Xenograft Model of AR-Negative Metastatic Castration-Resistant Prostate Cancer
Source: Cells. 2024 Apr 12;13(8):673. doi: 10.3390/cells13080673 (PMC11049137; doi:10.3390/cells13080673)
Supplement: Supplementary file 1 [file cells-13-00673-s001.zip › Figure S4 - revision.pptx]

## Slide 1
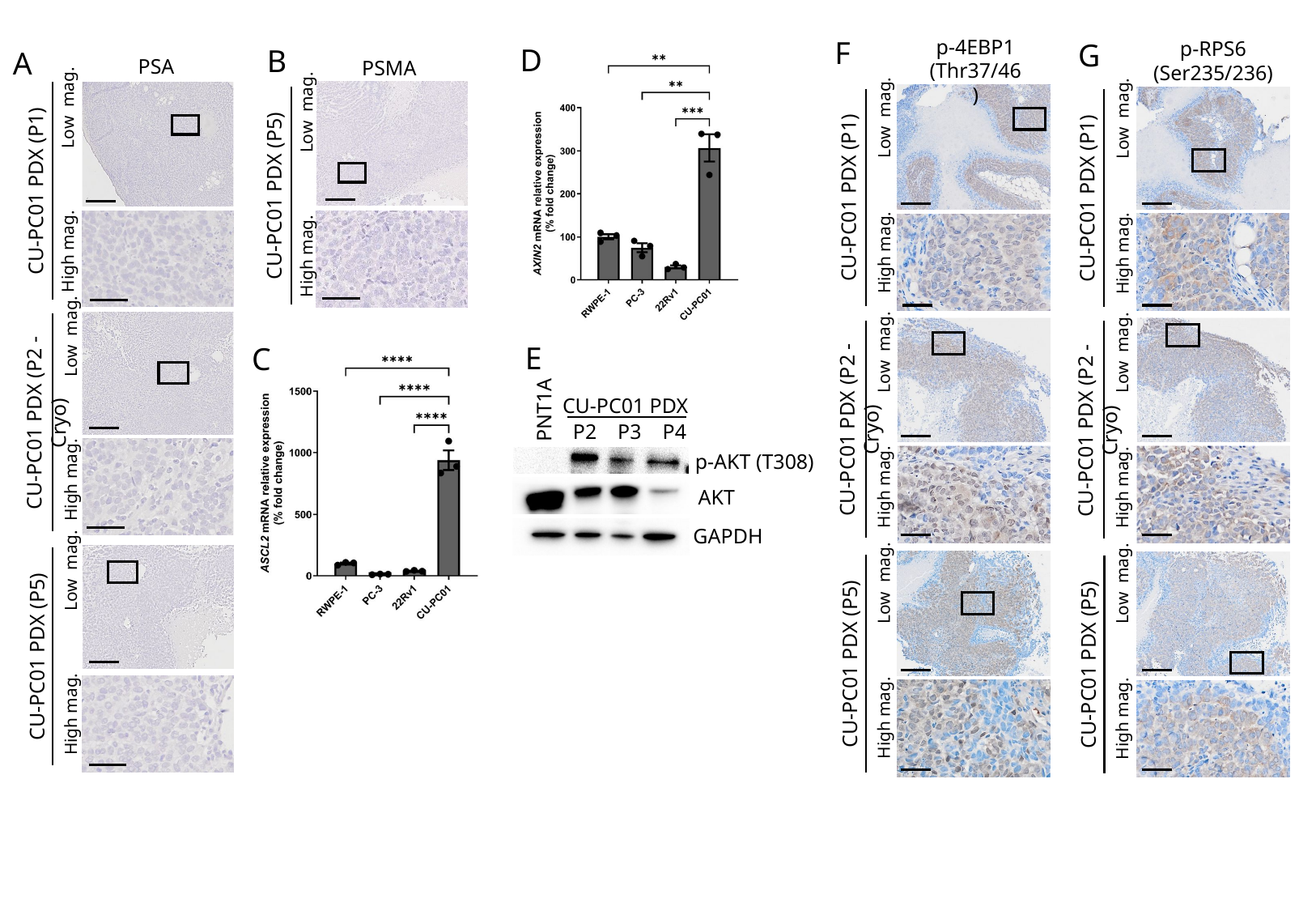

p-4EBP1 (Thr37/46)
F
G
p-RPS6 (Ser235/236)
D
B
A
PSA
PSMA
High mag. Low mag.
High mag. Low mag.
High mag. Low mag.
High mag. Low mag.
CU-PC01 PDX (P1)
CU-PC01 PDX (P5)
CU-PC01 PDX (P1)
CU-PC01 PDX (P1)
E
PNT1A
CU-PC01 PDX
P2 P3 P4
p-AKT (T308)
AKT
GAPDH
C
High mag. Low mag.
High mag. Low mag.
High mag. Low mag.
CU-PC01 PDX (P2 - Cryo)
CU-PC01 PDX (P2 - Cryo)
CU-PC01 PDX (P2 - Cryo)
High mag. Low mag.
High mag. Low mag.
High mag. Low mag.
CU-PC01 PDX (P5)
CU-PC01 PDX (P5)
CU-PC01 PDX (P5)
