# Supplementary material for: Development and Characterisation of a New Patient-Derived Xenograft Model of AR-Negative Metastatic Castration-Resistant Prostate Cancer
Source: Cells. 2024 Apr 12;13(8):673. doi: 10.3390/cells13080673 (PMC11049137; doi:10.3390/cells13080673)
Supplement: Supplementary file 1 [file cells-13-00673-s001.zip › Suppl Table 1.pdf]

Supplementary Table S1: Prostate cancer signalling pathway gene lists

Pathway	**AR signaling (n=22)[26-28]**	**MAPK signaling (n = 167)[30]**	**PI3K signaling (n = 68)[31]**	**NF-kB signaling (n = 48)[30, 32, 33]**	**NEPC-linked genes (n = 31)[35, 36]**	**Epigenetic regulators (n=12)[29]**	**Cell cycle (n=5)[29]**	**DNA repair (n=11)[29]**	**Wnt signaling (n=70)[34]**
**Genes**	*AR*	*ADORA2B*	*PIK3CA*	*REL*	*TP53*	*ARID1A*	*CCND1*	*ATM*	*FZD1*
	ARSE	*ADRA2B*	*PIK3CB*	*NFKB2*	*TTN*	*ARID2*	*CDK4*	*ATR*	*FZD2*
	CHRNA3	*AGT*	*PIK3CD*	*CYLD*	*DST*	*ARID4A*	*CDKN1B*	*BRCA1*	*FZD3*
	CRY1	*APOA1*	*PIK3R1*	*IKBA*	*MUC16*	*KDM6A*	*RB1*	*BRCA2*	*FZD4*
	CTCF	*ARHGEF6*	*PIK3R2*	*NEMO*	*ZFHX4*	*KMT2A*	*CDKN2A*	*CDK12*	*FZD5*
	ERG	*ATF1*	*PIK3R3*	*BCL3*	*ZNF479*	*KMT2C*		*CDH1*	*FZD6*
	ETV1	*ATF2*	*PIK3CG*	*IRAK4*	*CACNA1B*	*KMT2D*		*FANCA*	*FZD7*
	ETV4	*AVP*	*PIK3R5*	*TRAF6*	*ZNF99*	*MBD1*		*MLH1*	*FZD8*
	FKBP51	*BRAF*	*PIK3R6*	*RELA*	*CMYA5*	*SETD2*		*MSH2*	*FZD9*
	FOXA1	*BRAP*	*PIK3C2A*	*RELB*	*RYR1*	*SETDB1*		*MRE11A*	*FZD10*
	KLK2	*CAMKK2*	*PIK3C2B*	*EDA*	*OBSCN*	*SMARCA1*		*PALB2*	*LGR4*
	KLK3	*CAV1*	*PIK3C2G*	*PYRIN*	*RYR2*	*SMARCAD1*			*LGR5*
	KKL4	*CCL11*	*PIK3C3*	*NOD2*	*RB1*				*LGR6*
	KMO	*CCL2*	*PIK3R4*	*NOD1*	*AURKA*				*LRP5*
	LTBP1	*CCL3*	*PDPK1*	*TNFR1*	*MYCN*				*LRP6*
	NBPF3	*CCL5*	*AKT1*	*RANK*	*MSH6*				*RNF43*
	NCOA1	*CCM2*	*AKT2*	*SH2D1A*	*INSM1*				*ROR1*
	NCOA2	*CCR5*	*AKT3*	*TLR2*	*CDH2*				*ROR2*
	NCOA3	*CDC42EP5*	*RPS6KB1*	*TLR4*	*ASXL3*				*RYK*
	NKX3.1	*CDK1*	*RPS6KB2*	*FLT4*	*SPDEF*				*VANGL1*
	NR2E1	*CREB1*	*RPS6KB3*	*CASP12*	*EZH2*				*VANGL2*
	TMC8	*CRKL*	*SGK1*	*VHL*	*ASCL1*				*ZNRF3*
	TMPRSS	*CRYAB*	*SGK2*	*TLR3*	*NEUROD1*				*DKK1*
		CTSH	*SGK3*	*STAT6*	*BRN2*				*DKK2*
		DOK4	*AKT1S1*	*NFKB1*	*PEG10*				*DKK3*
		DOK5	*DEPTOR*	*TNFA*	*SRRM4*				*DKK4*
		DUSP10	*MTOR*	*FASL*	*SOX2*				*RSPO1*

DUSP19	*MLST8*	*COX2*	*ONECUT2*	*RSPO2*
DUSP28	*MAPKAP1*	*IL6*	*ZNF711*	*RSPO3*
DUSP3	*PRR5*	*IFNG*	*MYT1L*	*RSPO4*
DUSP6	*RPTOR*	*PAI1*	*MYT1*	*SFRP1*
DUSP7	*RICTOR*	*VWF*		*SFRP2*
DUSP9	*TELO2*	*CHUK*		*SFRP3*
EGF	*TTI1*	*FADD*		*SFRP4*
EGFR	*FOXO1*	*IKBKB*		*SFRP5*
ELK1	*FOXO3*	*IKBKG*		*WIF1*
EPGN	*FOXO4*	*IL1A*		*WNT1*
FGF10	*FOXO6*	*IL1R1*		*WNT2*
FGF13	*CAMKK2*	*MAP3K1*		*WNT2B*
FGF8	*MAP3K7*	*MAP3K7*		*WNT3*
FGFR1	*PRKAA1*	*MAP3K4*		*WNT3A*
FGFR3	*PRKAA2*	*MYD88*		*WNT4*
FOS	*PRKAB1*	*NFKBIA*		*WNT5A*
GPS1	*PRKAB2*	*RIPK1*		*WNT5B*
GPS2	*PRKAG1*	*TAB1*		*WNT6*
HRAS	*PRKAG2*	*TNFRSF1A*		*WNT7A*
HTR2B	*PRKAG3*	*TRADD*		*WNT7B*
IKBKG	*FKBP5*	*TNF*		*WNT8A*
IL18	*INPP5D*			*WNT8B*
IL1B	*INPPL1*			*WNT9A*
IL31RA	*INPP5J*			*WNT9B*
INS	*INPP4B*			*WNT10A*
IQGAP3	*PHLPP1*			*WNT10B*
IRAK1	*PHLPP2*			*WNT11*
IRAK2	*PPP2CA*			*WNT16*
IRAK4	*PTEN*			*APC*
ITGAV	*TSC1*			*AXIN1*

ITPKB	*TSC2*	*AXIN2*
JUN	*TBC1D7*	*BCL9*
KRAS	*STK11*	*CTNNB1*
LRRK2	*SESN1*	*DVL1*
MAP2K1	*SESN2*	*DVL2*
MAP2K2	*SESN3*	*DVL3*
MAP2K3	*RHEB*	*GSK3B*
MAP2K4	*RRAGA*	*PORCN*
MAP2K5	*RRAGB*	*PYGO1*
MAP2K6	*RRAGC*	*PYGO2*
MAP2K7	*RRAGD*	*TCF3*
MAP3K10		*TCF4*
MAP3K11		*TCF7*
MAP3K12		
MAP3K13		
MAP3K14		
MAP3K15		
MAP3K19		
MAP3K3		
MAP3K4		
MAP3K5		
MAP3K7		
MAP3K8		
MAP4K2		
MAP4K3		
MAPK1		
MAPK10		
MAPK11		
MAPK12		
MAPK13		

MAPK14
MAPK15
MAPK3
MAPK4
MAPK6
MAPK7
MAPK8
MAPK8IP2
MAPK9
MAPKAPK2
MAPKAPK3
MAPKAPK5
MED1
MEF2A
MEF2C
MEN1
MINK1
MOS
MT3
MYC
NDST1
NEK1
NF1
NLK
NOD1
NOD2
NPHS1
NRAS
NRG1
NRTN
OXTR

PAK1
PAK3
PLVAP
POU4F2
PPM1L
PPP2R5D
PTGER4
PTK2B
RAF1
RAPGEF2
RASGRP3
RB1CC1
RBM4
RET
RHBDD3
RIPK2
ROR2
RPS27A
RPS6KA1
RPS6KA2
RPS6KA3
RPS6KA5
SBK2
SCG2
SETX
SH2D3A
SH2D3C
SHANK3
SHC1
SMAD1
SOX9
STRADB
TAB1
TAB2
TAB3
TAOK1

TAOK2
TAOK3
TGFB1
TNF
TNFRSF19
TNFSF11
TRAF6
TRIB1
UBA52
UBB
UBC
YWHAB
