# Supplementary material for: Development and Characterisation of a New Patient-Derived Xenograft Model of AR-Negative Metastatic Castration-Resistant Prostate Cancer
Source: Cells. 2024 Apr 12;13(8):673. doi: 10.3390/cells13080673 (PMC11049137; doi:10.3390/cells13080673)
Supplement: Supplementary file 1 [file cells-13-00673-s001.zip › Suppl Table 4.pdf]

Supplementary Table S4: Frequency of genetic alterations in NEPC patient specimens for all conserved genetic variants detected in CU-PC01 PDX tumors[‡]

Gene	% Frequency (n, patients)	Type (n observed)
**AR signaling**		
AR	2.2 % (1/45)	Amplification (1)
ARSE (ARSL)	0 % (0/45)	
CHRNA3	2.2% (1/45)	Truncation: E393* (1)
ETV1	2.2% (0/45)	Amplification (1)
LTBP1	2.2% (1/45)	X1612_splice (1)
NBPF3	8.9% (4/45)	Missense (4)
TMC8	4.4% (2/45)	Truncation: K328* (1)<br>Missense (1)
**NF-κB signaling**		
MAP3K1	2.2 % (1/45)	Homozygous deletion (1)
MAP3K4	2.2% (1/45)	Missense (1)
IL6	0 % (0/45)	
VWF	0 % (0/45)	
**Epigenetic regulators**		
KMT2D	6.7% (3/45)	Missense (3)
**NEPC-associated genes**		
TP53	44.4% (20/45)	Missense (9)<br>Truncation (6)<br>Splice (2)<br>Homozygous deletion (3)
TTN	20.0% (9/45)	Missense (9) ł<br>Truncation: E5206* (1)
DST	11.1% (5/45)	Missense (6) ł
MUC16	15.6% (7/45)	Missense (9) ł
ZFHX4	6.7% (3/45)	Missense (3)
ZNF479	8.9% (4/45)	Missense (4)
CACNA1B	8.9% (4/45)	Missense (4)
CMYA5	11.1% (5/45)	Missense (5)
OBSCN	4.4% (2/45)	Missense (4) ł
RYR2	6.7% (3/45)	Missense (4) ł
AURKA	2.2 % (1/45)	Amplification (1)
ASXL3	0 % (0/45)	
NEUROD1	0 % (0/45)	
SRRM4	0 % (0/45)	
ASCL1	0 % (0/45)	
EZH2	0 % (0/45)	
**DNA damage repair**		
ATM	2.2 % (1/45)	Missense (1)
BRCA2	13.3% (6/45)	Missense (2)<br>Truncation (2)<br>Homozygous deletion (2)
**PI3K signaling**		
INPP5D	0 % (0/45)	

PIK3CA	4.4% (2/45)	Missense: G106R (1) = GOF<br>Truncation: R4* (1)
PIK3CG	0 % (0/45)	
PIK3R2	2.2% (1/45)	Missense (1/45)
PIK3R3	2.2% (1/45)	Missense (1/45)
RICTOR	0 % (0/45)	
**Wnt signaling**		
FZD6	0 % (0/45)	
LGR6	0 % (0/45)	
RSPO2	0 % (0/45)	
SFRP1	0 % (0/45)	
APC	6.7 % (3/45)	Truncation (4) = LOF ƚ<br>Homozygous deletion (1)<br>X113_Splice + Homozygous deletion (1)
BCL9	0 % (0/45)	
CTNNB1	4.4% (2/45)	Missense (2/45) = GOF
RNF43	4.4 % (2/45)	Truncation (2) ƚ<br>Amplification (1)

ǂ Data was sourced from NEPC patient whole exome mutation data entered into cBioPortal.org (accessed January 2024) from the prostate cancer neuroendocrine multi-site study (Beltran et al, Nat med, 2016, mutation data only, n = 30 patients, n = 44 samples) and neuroendocrine and small cell neuroendocrine patients in the prostate cancer MSK dataset (Abida et al, JCO Precis Oncol 2017, mutation and CNV data, n = 15 patients, n = 17 samples). In total, n = 45 patients and n = 61 samples with mutational data for 100% (61/61) of the samples, and CNV data for just 27.9% (17/61) of the samples analysed. GOF = Gain of function mutation; LOF = loss of function mutation.

ƚ One or more patients carried more than one alteration, either in the same sample, or across multiple samples collected from the same patient.
