# Supplementary material for: Development and Characterisation of a New Patient-Derived Xenograft Model of AR-Negative Metastatic Castration-Resistant Prostate Cancer
Source: Cells. 2024 Apr 12;13(8):673. doi: 10.3390/cells13080673 (PMC11049137; doi:10.3390/cells13080673)
Supplement: Supplementary file 1 [file cells-13-00673-s001.zip › Supplemental material.pdf]

Supplemental material

<u>Figure legends</u>

Figure S1. Histopathological analysis of CU-PC01 PDX tumours revealed phenotypic preservation across serial passages and post-cryopreservation. (A) Plot displays CU-PC01 tumour volume at passage 2 (P2), and post-cryopreservation (P2-cyro), revealing cryopreservation slightly reduces tumour growth rate (n = 3). Representative H&E images of CU-PC01 PDX tumour specimens harvested from (B) NSG mice at passage 2-6, (C) Athymic nude mice at passage 7 and 8 and (D) NSG mice engrafted with a cryo-preserved CU-PC01 passage 2 tissue fragment. Scale bar = 50 μm.

Figure S2. The mutational landscape of the donor patient biopsy is well-conserved in the CU-PC01 PDX tumor model across multiple passages. (A) Whole exome sequencing analysis to determine the mutational frequency of mutated genes in the patient biopsy, passage 1 (P1) and passage 5 (P5) CU-PC01 PDX tumours engrafted into NSG mice, and passage 7 (P7) PDX tumours engrafted into athymic nude mice. A high concordance between the mutational landscape of the original patient biopsy and the PDX tumours in both NSG and athymic nude immunocompromised mice was observed. (B) Agarose gel image displays PCR product analysis of gDNA isolated from the patient lymph node biopsy and CU-PC01 PDX tumours (passage 1-8, P1-8) (n = 3 independent repeats). The absence of a positive band in CU-PC01 PDX tumours confirmed EBV type1/2 lymphoma is not present in these tumours, consistent with mCRPC. Negative (-ve) control = gDNA isolated from the LNCaP human prostate carcinoma cell line. Positive (+ve) control = gDNA isolated from the Raji cell line, where 801 bp product of *EBNA2* detects EBV type-1 and type-2. M= molecular weight marker (100 bp ladder).

Figure S3. CU-PC01 PDX tumours display RB loss, weak HOXB13 expression and lack several NE markers. (A) Representative RB IHC images from CU-PC01 PDX tumours at passage P1, P2-cryo and P5 and (B) PC-3 subcutaneous xenograft tumor (positive control) (n = 3). (C) Western Blotting to detect RB (110-116 kDa) and GAPDH (37 kDa) in PC-3 xenograft (positive control) and CU-PC01 PDX (passage 2) tumours (n = 3), confirming RB is lost in the CU-PC01 PDX model (n=3 independent repeats). (D) Representative IHC images from CU-PC01 PDX tumours (P5 shown) for HOXB13, ASCL1, NEUROD1 and POU2F3. Low magnification scale bars = 200 μm, high magnification scale bars = 50 μm.

Figure S4. The CU-PC01 PDX model does not express PSA or PSMA and displays elevated Wnt and PI3K signalling. (A) Representative PSA IHC images from CU-PC01 PDX tumours at passage P1, P2-cryo and P5. (B) Representative PSMA IHC image from CU-PC01 PDX tumours (P5 shown). QRT-PCR analysis of CU-PC01 tumours (P2) for (C) *ASCL2* and (D) *AXIN2* mRNA transcripts normalized to GAPDH reveal an increase in expression relative to human RWPE-1 non-malignant prostate epithelial cells and malignant PC-3 and 22Rv1 human mCRPC cell lines that do not carry a Wnt driver mutation. Error bars = S.E.M (n = 3, 3 independent repeats). One-Way ANOVA with Tukey correction, ** $P \leq 0.0045$, *** $P = 0.0005$, **** $P < 0.0001$. (E) Western Blotting to detect p-AKT T308 (60 kDa), total AKT (60 kDa) and GAPDH (37 kDa) in CU-PC01 PDX tumours at passage P2, P3 and P4 (n = 3). Protein lysates from non-malignant PNT1A human prostate epithelial cells served as a negative control for p-AKT. Representative IHC images from CU-PC01 PDX tumours at passage P1, P2-cryo and P5 to detect (F) p-4EBP1 and (G) p-RPS6 revealed a high abundance of both phosphorylated PI3K/AKT pathway downstream substrates (n = 3). Low magnification scale bars = 200 μm, high magnification scale bars = 50 μm.

Figure S5. Enzalutamide and docetaxel treatment has no effect on CU-PC01 PDX tumor burden. Plot shows CU-PC01 PDX tumor weight from athymic nude mice that were subcutaneously implanted with CU-PC01 PDX tumor fragments before being treated with either vehicle, enzalutamide (10 mg/kg p.o. 5

days on 2 days off) or docetaxel (10 mg/kg i.p once a week) for 15 days. Error bars represent S.E.M, one-way ANOVA with Tukey correction revealed no significant difference between treatment arms (P-value >0.05, n = 4-6/treatment arm).

Table legends

Table S1. Gene list of signaling pathways and cellular processes that are commonly altered in prostate cancer. Table displays the gene lists employed to explore signalling pathways that are commonly deregulated in prostate cancer.

Table S2. CU-P01 PDX model whole exome sequencing data summary. Table displays all SNPs and INDELs observed in the donor patient lymph node biopsy and PDX tumours collected at passage 1, 5 and 7.

Table S3. CU-P01 PDX model allelic frequency data summary. Table displays the allelic frequency (AF) for all SNPs and INDELs observed in the donor patient lymph node biopsy and CU-PC01 PDX tumours collected at passage 1, 5 and 7 according to the chromosome and nucleotide position. Heterozygous AF = 0.50 and Homozygous AF = 1.00.

Table S4. Frequency of genetic alterations in NEPC patient specimens for all conserved genetic variants detected in CU-PC01 PDX tumours. Table displays the frequency of genetic aberrations according to publicly available NEPC datasets for genes in the CU-PC01 PDX model that carried conserved genetic variants. ƚ Data was sourced from NEPC patient whole exome mutation data entered into cBioPortal.org (accessed January 2024) from the prostate cancer neuroendocrine multi-site study (Beltran et al, Nat med, 2016, mutation data only, n = 30 patients, n = 44 samples) and neuroendocrine and small cell neuroendocrine patients in the prostate cancer MSK dataset (Abida et al, JCO Precis Oncol 2017, mutation and CNV data, n = 15 patients, n = 17 samples). In total, n = 45 patients and n = 61 samples with mutational data for 100% (61/61) of the samples, and CNV data for just 27.9% (17/61) of the samples analysed. GOF = Gain of function mutation; LOF = loss of function mutation. ƚ One or more patients carried >1 alteration, either in the same sample, or across multiple samples collected from the same patient.
