# Supplementary figures and images for: Development and Characterisation of a New Patient-Derived Xenograft Model of AR-Negative Metastatic Castration-Resistant Prostate Cancer
Source: Cells. 2024 Apr 12;13(8):673. doi: 10.3390/cells13080673 (PMC11049137; doi:10.3390/cells13080673)

## Slide 1
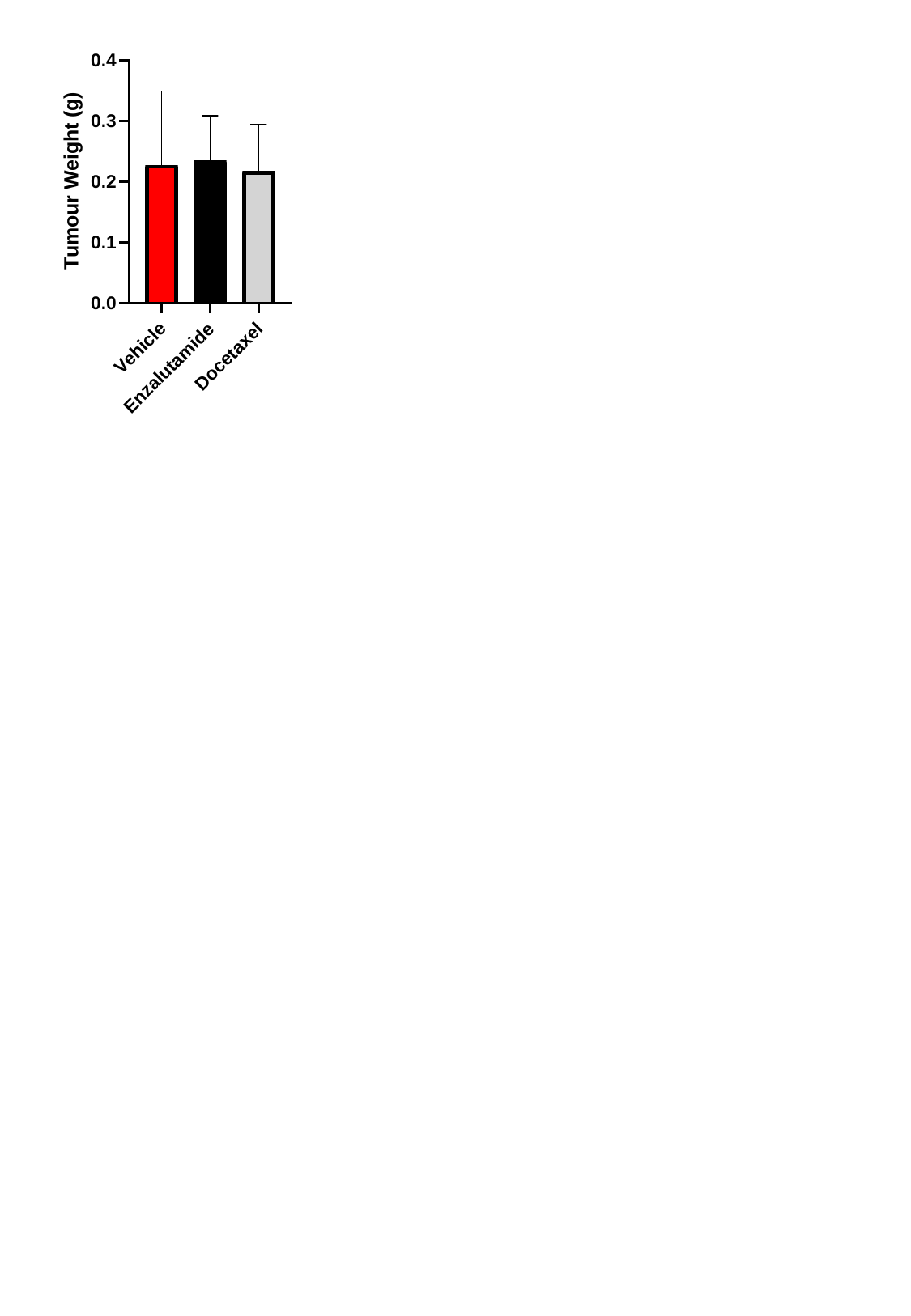

Supplement: Supplementary file 1 [file cells-13-00673-s001.zip › Figure S5 - revision.pptx]
